# Origins Under the Blowtorch: Frequent Fire Shifts the Balance Between Sunda‐Origin and Sahul‐Origin Plant Species in a Tropical Savanna

**DOI:** 10.1002/ece3.73837

**Published:** 2026-06-21

**Authors:** Susanna Rozsa Bryceson, John William Morgan

**Affiliations:** ^1^ Department of Ecological, Plant & Animal Sciences La Trobe University Bundoora Victoria Australia

## Abstract

Modern savanna fire management is based on climatic season and fire frequency. However, the different biogeographic origins of savannas across the world influence their ecosystem functioning, making them floristically highly dissimilar. We examine the effect of fire frequency on the composition of vegetation of different biogeographic origins, to understand how frequently an ecosystem can be subject to fire but still retain its evolutionary diversity. We surveyed savanna subject to a long‐term fire‐frequency experiment in Darwin, Australia, and analysed vegetation changes according to species' biogeographic origin as either Sunda (Southeast Asia) or Sahul (ancient Australia and New Guinea). We found dramatic structural and biogeographic change in less than 20 years of frequent burning. Vegetation transformed from dry tropical woodlands into savannas, with the lens of biogeographic origin revealing deeper trends. Plots subject to fire every 1–3 years caused a shift from mostly Sahul‐origin, multi‐strata vegetation into simple tree‐grass systems dominated by Sunda‐origin Andropogoneae annual grasses, with ramifications for all Sahul‐origin taxa. Despite the common physiognomy of the world's savannas, no single fire‐frequency regime suits all—local ecosystem composition and dynamics need to underpin all prescribed burning regimes. In northern Australia, fire management that ultimately promotes the shift to Sunda‐origin grasses threatens the continued existence of ancient Sahul‐origin plants and animals which had not evolved with grass‐fire cycles. We call for inclusion of species origin in analyses of ecosystems wherever modern and ancient elements cohabit. In a policy and management sense, the promotion of annual grasses by frequent fire also affects the management of savannas.

## Graphical abstract

We examined the effect of fire frequency on the composition of vegetation of different biogeographic origins, to understand how frequently an ecosystem can be subject to fire but still retain its evolutionary diversity. There was dramatic structural and biogeographic change in less than 20 years from dry tropical woodlands into savannas, creating intense fires and threatening the continued existence of ancient Sahul‐origin plants and animals which had not evolved with frequent grass‐fire cycles.

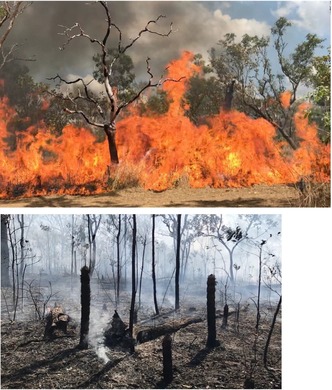

## 1 Introduction

Savannas comprise two contrasting growth forms: scattered trees and a continuous understorey of flammable C_4_ grasses (Parr et al. 2014). They evolved from the mid‐Miocene (c15 Ma) supporting complex biomes characterised by grazing animals, carnivores, and scavengers in the tropics of Africa, Eurasia, and the Americas (Kaya et al. 2018; Stromberg 2011).

Fire is integral to savanna sustainability; without it, woody plant cover increases and the structural openness required for their biodiversity is reduced (Parr et al. 2014). *Prima facie*, the ecological dynamics of a ‘true’ savanna experience are activated by ‘natural fire’, that is, as sparked by lightning; however, there is evidence of hominids using fire in African savannas more than a million years ago and, in contemporary savannas, most fires are ignited by humans (Archibald 2016; Russell‐Smith et al. 2007). In tropical northern Australia, savannas do not appear to be lightning‐associated although lightning activity is relatively high (Kuleshov et al. 2006)—only a small proportion of strikes ignite fires because storms often bring rainfall and many strikes occur on non‐flammable surfaces. Around 80%–90% of fires occur before the main lightning storm period and, as such, are likely to be human‐ignited (Murphy et al. 2010; Russell‐Smith et al. 2007).

In pre‐colonial landscapes, over tens of thousands of years, Aboriginal people used fire for many purposes—including hunting, plant management, signalling, cultural activities, and maintaining open areas around camps, waterholes and travel routes—typically lighting small fires early in the dry season (Bliege Bird et al. 2008; Bowman and Prior 2004; Russell‐Smith et al. 2007). Today, fire is the main land management tool in the Australian monsoon tropics and northern savanna country, used for clearing shrubs for grazing livestock and to reduce fuel for severe late‐season fire (Bowman 1998; Prober et al. 2016; Russell‐Smith et al. 1997).

Modern fire management is based on two key aspects: climatic season and fire frequency. To prevent damaging fires during the late dry‐season, igniting ‘cool season fires’ in the early dry season (EDS) is now a common practice in savannas in Africa (Archibald 2016), Madagascar (Vorontsova et al. 2016), South America (Moreira Santos et al. 2022) and northern Australia (Russell‐Smith et al. 2020), where it is often linked to traditional Aboriginal methods. These approaches underpin large‐scale carbon abatement programs and conservation management strategies across much of the northern Australian savannas. However, the ecological consequences of these interventions remain contested, particularly regarding their implications for biodiversity and their relationship to historical Indigenous fire regimes (Bowman et al. 2020; Corey et al. 2020).

Fire frequency is more complex. Long‐term experiments—in Africa since the 1950s (van Wilgen et al. 2022) and northern Australia since the 1970s (Andersen et al. 2005)—compare unburned areas to treatment sites in which fire is applied repeatedly. Their findings are largely similar: frequent fire removes the shrub layer, creates systems dominated by immature plants, promotes C_4_ grasses and kills fire‐sensitive plants. However, the different biogeographic origins of savannas across the world influence their ecosystem functioning (Edwards et al. 2010), making them floristically highly dissimilar (Bryceson and Morgan 2022) and, despite their common physiognomy, there is no single fire regime that suits every situation (Parr and Andersen 2006). The current distributions of grassy biomes reflect a confluence of abiotic factors, evolutionary history and human‐mediated disturbance (Lehmann et al. 2014).

Australia stands apart. Compared to the formerly conjoined Americas‐Africa‐Eurasia conglomerate, the evolutionary journey of the Papua New Guinea‐Australian continent (‘Sahul’) is distinctive because of the long isolation of its ancient taxa (Crisp and Cook 2013). As Sahul neared Asia due to continental drift, the influx of lowland and savanna plants from tropical ‘Sunda’ (Southeast Asian continental shelf) during the Pleistocene (Crayn et al. 2015), along with the dispersal of Andropogoneae grasses in the past 1 Ma, introduced disturbance dynamics on a scale previously not experienced by Sahul's highly niche‐conserved flora and fauna (Bryceson et al. 2023). Today, Australian savannas occupy vast tracts of the monsoon tropics, forming tree‐grass systems on low‐plains, interspersed with rocky sandstone uplands (Bowman et al. 2010).

While climate and environmental conditions can define the fundamental ecological niche of a species, it is often the historical components of ecological puzzles—such as an organism's genetic history, its continent's geological history and its ecosystem's human history—that ultimately explain where a species *actually* occurs in contemporary landscapes (Estes and Vermeij 2022). In the spirit of Stebbins and Major (1965), this study takes a biogeographic approach to analysing the effects of fire regimes on Australian tropical savanna, adding another piece to the puzzle of savanna management. The co‐occurrence of plants with origins from Sunda and Sahul makes it an ideal place to test relationships between fire frequencies and species of different biogeographic origins.

We used a long‐term fire regime experiment in the mesic savanna of the Australian monsoon tropics to understand the links between fire frequency, fire season and origin of plants in the resultant vegetation composition. In particular, we asked.

- What effect does fire frequency have on the composition of vegetation relative to its biogeographic origin?
- How frequently can an ecosystem be subject to fire but still retain its evolutionary diversity?

## 2 Methods

### 2.1 Study Site

The vegetation survey was carried out at the CSIRO ‘Burning for Biodiversity’ experimental site at Berry Springs, 50 km south of Darwin, Northern Territory Australia (12° 41′ 42.25″ S, 130° 58′ 50.36″ E). The site lies in the Australian monsoon tropics: mean annual precipitation is 1401 mm, with only 10% occurring between April and November; mean monthly minimum temperature is 20.9°C; and mean monthly maximum is 33.1°C (Berry Springs Station, station no. 14264, www.bom.gov.au). Soils are relatively uniform brown sandy loams or clay loams, derived from weathered sandstone, siltstone and shale, and ranging from 0.5 to 1.0 m in depth, increasing towards the south of the experiment site. The site is underlaid by a laterite horizon, varying from 58 to 114 cm below the soil surface (Scott et al. 2012). Soil moisture at the southern end of the site was higher due to its proximity to an ephemeral wetland. Mean soil pH was between 4.5 and 4.8, depending on fire treatment (Blunden et al. 2024).

### 2.2 Land‐Use History

The tropical northern part of the Northern Territory is known as the ‘Top End’. There is evidence of human occupation here more than 50,000 years ago (Florin et al. 2020) and Aboriginal people managed the lands in this area until the 1870s, when European settlers brought livestock to the region and the first pastoral leases were issued in the north of Adelaide River. Low‐density cattle grazing persisted until the 1950s, when cattle production expanded across the Berry Springs district and the Top End generally. Stock moved through largely unfenced country, grazing native grasses and travelling along major cattle routes connecting stations with markets in Darwin (McLaren and Cooper 2001).

The site for the Territory Wildlife Park (TWP) was selected because it lies close to freshwater and its vegetation was largely unaffected by Cyclone Tracey in 1974. A perimeter fence was constructed in 1986 and the 400‐ha park opened to the public in 1989 (Northern Territory Government 2026). Wetlands to the east and south separate the research site from the main visitor area. Unlike the surrounding region which is regularly burned for fuel reduction, TWP is managed largely through fire exclusion, with a maintained perimeter firebreak. Consequently, the site has experienced little fire since 1989, apart from burns in the A plots in 1993 and 2000 and in the B and C plots in 1992 (Scott et al. 2012). Such long‐unburned vegetation is now uncommon in the monsoon tropics.

### 2.3 Recent Vegetation History

The Commonwealth Scientific and Industrial Research Organisation (CSIRO) began the ‘Burning for Biodiversity’ experiment in 2004. Vegetation surveys at the start of the experiment showed clear differences among plots in this woodland site. The southern C plots had substantially greater canopy cover and species richness than the A and B plots, with more shade and leaf litter and relatively few grass species, dominated by the perennial *Eriachne triseta* (Scott 2012). The A and B plots were broadly similar, with a more open mid‐storey and a greater grass component. They were dominated by the perennials *Eriachne triseta* and *E. avenacea*, and the annual *Pseudopogonatherum contortum*, together with other perennial grasses including *Sarga plumosum*, *Chrysopogon latifolius* and *Schizachyrium fragile*, and several low‐growing annual grass species (Scott 2012). The annual *Sarga intrans* occurred only sparsely within one A plot but formed dense stands immediately outside the western and northern fences.

At the time of our study, the site comprised mixed open forest and woodland *sensu* Specht (1970) with a tall overstorey dominated by *Eucalyptus tetrodonta*, *E. miniata* and *Corymbia bleeseri*. Lower canopies commonly sustained *Syzygium* and *Acacia* species, *Terminalia ferdinandiana*, *Buchanania obovata*, *Livistona humilis*, *Pandanus spiralis*, *Planchonia careya*, *Petalostigma quadriloculare* and *Calytrix exstipulata*. Understorey annual and perennial grasses included species of *Eriachne*, *Sarga*, *Pseudopogonantherum* and *Chrysopogon*. See Appendix S1 for a full species list.

### 2.4 Experimental Design

The experiment comprised three replicate blocks of six 1‐ha plots. Six fire treatments were applied in each block: early dry season (June)—yearly (E1); every 2 years (E2); every 3 years (E3); every 5 years (E5); late dry season (October)—every 2 years (L2); and unburned (UB). Fire treatments were randomly assigned at the start of the experiment and have been maintained since. All fires were conducted by igniting plot edges by strip‐burning, allowing head‐fires to form and burn through the plot proper. Flame height varied from very low (30 cm) to high (> 10 m).

### 2.5 Vegetation Survey

This study was based on vegetation surveys of all 18 plots at the site. The vegetation survey was carried out in contiguous 10 × 10 m quadrats in the each of the plots, positioned at least 20 m from the plot boundary to minimise edge effects. There were 27–36 quadrats per plot (site *n* = 573) (see Appendix S2 for quadrat details). Surveys were conducted in June 2019 and 2021 in the week before the ‘early season’ prescribed burns, to record plants at maximum size before the fires (with the exception that the L2 plots were burned 4 months after the June 2019 survey). The survey recorded plants which may be associated with fire dynamics and physiognomy, namely, all woody species, grass species, and sprouts across the site. Species represented by single individuals were excluded from the analysis.

The vegetation survey recorded all species observed rooted in each quadrat. Vegetation was categorised according to height rather than DBH to incorporate a better sense of savanna physiognomy. DBH is commonly related to age, but we were more interested here in vegetation complexity. Further, DBH is a less useful indicator for shrubs, and to maintain uniformity across all woody vegetation, we recorded height and number of individuals to create a clear picture of the number of strata and vegetation complexity. The number of individuals of each woody plant species in each quadrat was recorded in four strata: trees > 8 m, small trees 3–8 m; shrubs 1–3 m, and sprouts to 1 m (resprouts of woody species). Grasses were recorded by estimating percent cover by each species in each quadrat. Per cent cover of bare ground and leaf litter was also recorded.

Vegetation structure was used as a proxy for shade cover, coded after Specht (1970) and cross‐checked against shade cover (%): 5 = Dense, crowns interlaced, > 75% shade cover; 4 = Mid‐dense, crowns touching, 50%–75% shade cover; 3 = Mid‐sparse, 1 crown‐width separation, 25%–50% shade; 2 = Sparse, > 1 crown‐width separation, 10%–25% shade; 1 = Very sparse, > 2 crown‐widths separation, < 10% shade.

### 2.6 Life Strategy

All trees, shrubs and sprouts recorded in this study were perennial species. With some grasses, life‐strategy can vary within a single species across its distribution, depending on growing conditions, so for this study, they were designated according to Flora of Australia and Atlas of Living Australia and verified in the field by the ease with which they could be pulled out of the ground by hand. Two *Eriachne* species listed as ‘ephemeral or annual’ were designated in this study as ‘annual’. One *Aristida* species was listed as ‘annual in dry regions, perennial’ in wetter tropical areas, so here it was designated as ‘perennial’. Three other species were designated ‘annual’: *Mnesithea formosa*, *Sarga intrans* and *Thaumastochloa major*.

### 2.7 Biogeographic Analysis

All plants were identified to genus level, and most to species level. All genera were sorted into ‘Sahul’ (PNG‐Australia continental shelf) or ‘Sunda’ (Southeast Asian continental shelf) categories according to their likely origin, derived from molecular phylogenies or dated phylogenetic trees wherever possible (see Appendix S1 for full list). Genera with cosmopolitan distributions were included in the ‘Sunda’ category as dispersal would have most likely have occurred through Southeast Asia (Crayn et al. 2015; Yap et al. 2018).

### 2.8 Statistical Analysis

To analyse shifts in vegetation composition in relation to fire frequency, each stratum was calculated separately for each treatment, and for each origin type (Sunda or Sahul). Mean plot abundance was calculated from the number of individuals of each species in each quadrat. The three replicate plot means were used to calculate treatment means (with associated standard error). Mean species richness was calculated for each treatment similarly. Data was analysed using R (R Core Team 2013) according to strata and treatment type by comparing means to identify trends and species richness (ANOVA). *Post hoc* Tukey HSD analyses identified differences between statistically significant groups.

To analyse structural composition, plot and treatment means were transformed into proportions of total vegetation. Trends in mean abundance of Sahulian and Sundanian species in each stratum were compared, and plot means were converted into proportions to investigate whole‐of‐site species‐origin trends. Interpretation of results followed the principles of ‘inference by eye’ using confidence intervals (margin of error) (Cumming and Finch 2005).

## 3 Results

### 3.1 Whole‐Of‐Site Vegetation Composition

In woody plants, 19 Sahulian genera and 23 Sundanian genera were included in this study. Of grasses, 13 genera were included and all used C_4_ photosynthesis. One genus was of Sahul‐origin (*Eriachne*), and the other 12 genera were Sunda‐derived, 8 from the Andropogoneae subtribe (see Appendix S1a for full species list).

**FIGURE 1:**
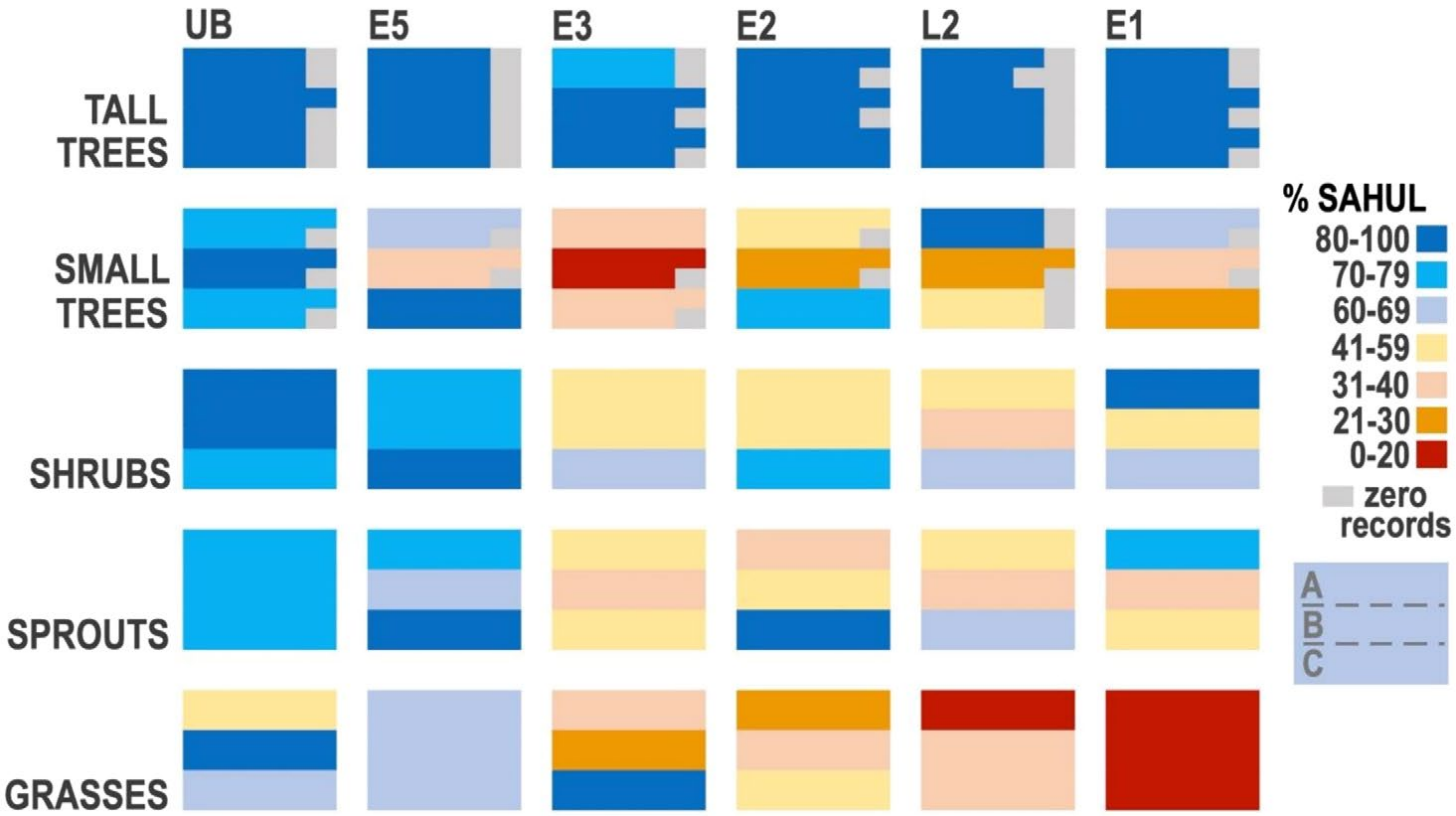
Vegetation survey showing mean proportion of Sahul‐origin plants across vegetation strata in relation to fire frequency, Berry Springs, Australia. The colour bands show mean proportions of Sahul‐origin plants vs. Sunda‐origin plants in each replicate plot (A, B and C) at each fire frequency. Fire frequency is designated as Early season (E) or Late (L), followed by numeral indicating years between fires. UB = Unburned. 100 m^2^ quadrat *n*: UB = 93; E5 = 90; E3 = 102; E2 = 91; L2 = 98; E1 = 99. The proportion shown for each replicate is a mean of all of its 10 × 10 m survey quadrats (*n* = 27–34). The proportion of plots that had no records at all for the vegetation type is shown in grey. For example, in Tall Trees E1, 20% of the A plots had no tall trees recorded, nor did 10% of both the B and C plots. The remaining Tall Trees E1 plots had a mean of 80%–100% Sahul‐origin plants.

When fire regime treatments (frequency, season) are viewed stratum by stratum (Figure 1), it is clear that Unburned (UB) and Early burn, 5‐year interval (E5) plots are characterised by Sahul‐origin species at every vegetation level, from the upper‐storey tree layers to the mid‐storey shrubs and the shorter sprout and grass layers. They are completely different, in terms of biogeographic origin, from the Late season 2‐year interval (L2)‐Early season 1‐year interval (E1) plots, apart from the tallest tree types. In the tall tree layer, Sahul‐origin species comprised more than 70% of species recorded across all fire frequencies. This pattern was not repeated in any of the other strata. In the small trees, the shift to Sundanian species occurs with 5‐year fire frequency, with a complete shift in dominance in the early season, 3‐year interval (E3), while in shrubs and sprouts, the proportions of Sahul‐Sunda were more even from E3 to E1. The pattern in grasses was dramatic: the shift to dominance by Sundanian species occurred in the E3 plots and increased with fire frequency. Annually burned plots are fully characterised by Sunda‐origin grass species.

### 3.2 Species Origin of Separate Strata

**FIGURE 2:**
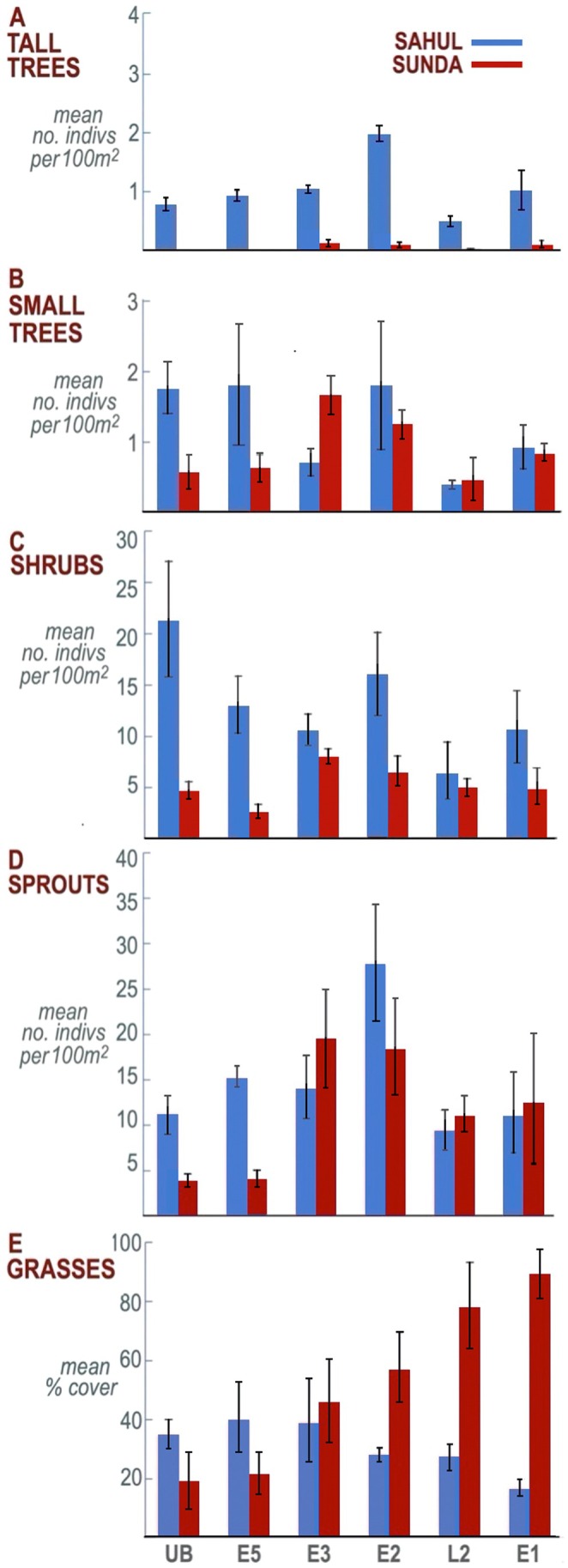
Comparison of Sahul and Sunda species abundance (mean ±1 SE) across vegetation strata in relation to fire frequency, Berry Springs, Australia. (a): Tall trees = plants > 8 m. (b): Small trees = plants 3–8 m. (c): Shrubs = plants 1–3 m. (d): Sprouts = plants < 1 m tall. (e): Grasses. Note ‘grass’ is recorded by % cover of the ground, whereas ‘shrubs’ and ‘trees’ represent individual plants. Fire frequency is designated as Early season (E) or Late (L), followed by numeral indicating years between fires. UB = Unburned.

**FIGURE 3:**
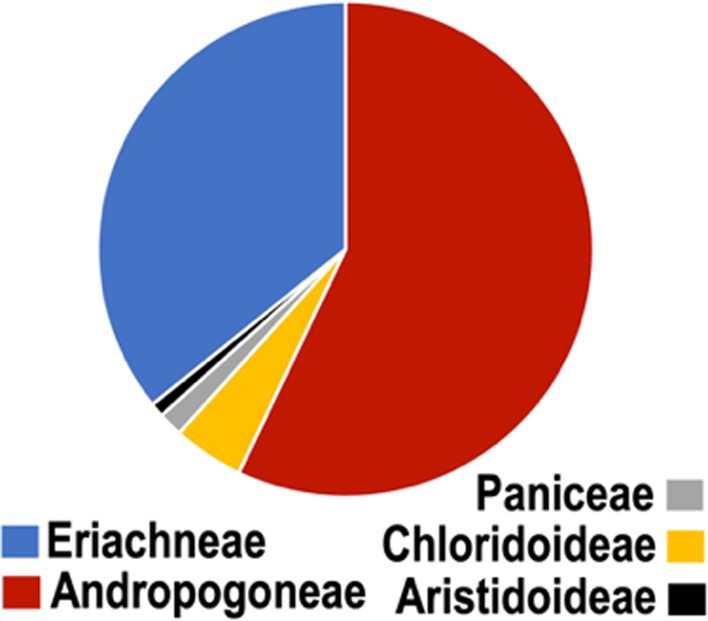
Composition of grasses across the site according to phylogenetic group. Sahulian grasses = Eriachneae. Sundanian grasses = Andropogoneae, Paniceae, Chloridoideae and Aristidoideae.

***Grasses*** became a steadily greater component of plots with every increase in fire frequency (Figure 2e). Grass cover across the site was dominated by species of two groups: the Sahulian tribe Eriachneae and Sundanian tribe Andropogoneae (Figure 3).

There was a significant difference between grass life strategies, with perennial grasses recording higher responses to treatments than annuals (ANOVA: *F* = 32.59, df = 1, *p* < 0.001). Sundanian grasses showed more variation across treatments than Sahulian, which remained comparatively stable. Treatment effects per se were not statistically significant; however, the combination of treatment effects with biogeographic origin was significant (ANOVA *F* = 3.61, df = 5, *p* = 0.007) (see Appendix S3 for ANOVA details).

Treatment effects were weakest in annual and perennial Sahulian grasses, and also in annual Sundanian grasses. In perennial Sundanian grasses, variation across treatments was marginally significant (*F* = 2.62, df = 5, *p* = 0.08), with the greatest change in the E5 treatment plots. These grasses had high coverage in E1 and L2 plots with low coverage in E5 and UB (see Appendix S3).

Block effects were generally weak across the dataset but were significant for Sundanian annual grasses. While they recorded no effect *across* treatments (*p* = 0.59), variation among blocks explained a significant portion of their occurrence across the site, where occurrence differed among blocks (*F* = 5.06, df = 2, *p* = 0.021). *Post hoc* comparisons indicated that these grasses had significantly more coverage in Block A than in Blocks B and C, which differed little (see Appendix S3).

**FIGURE 4:**
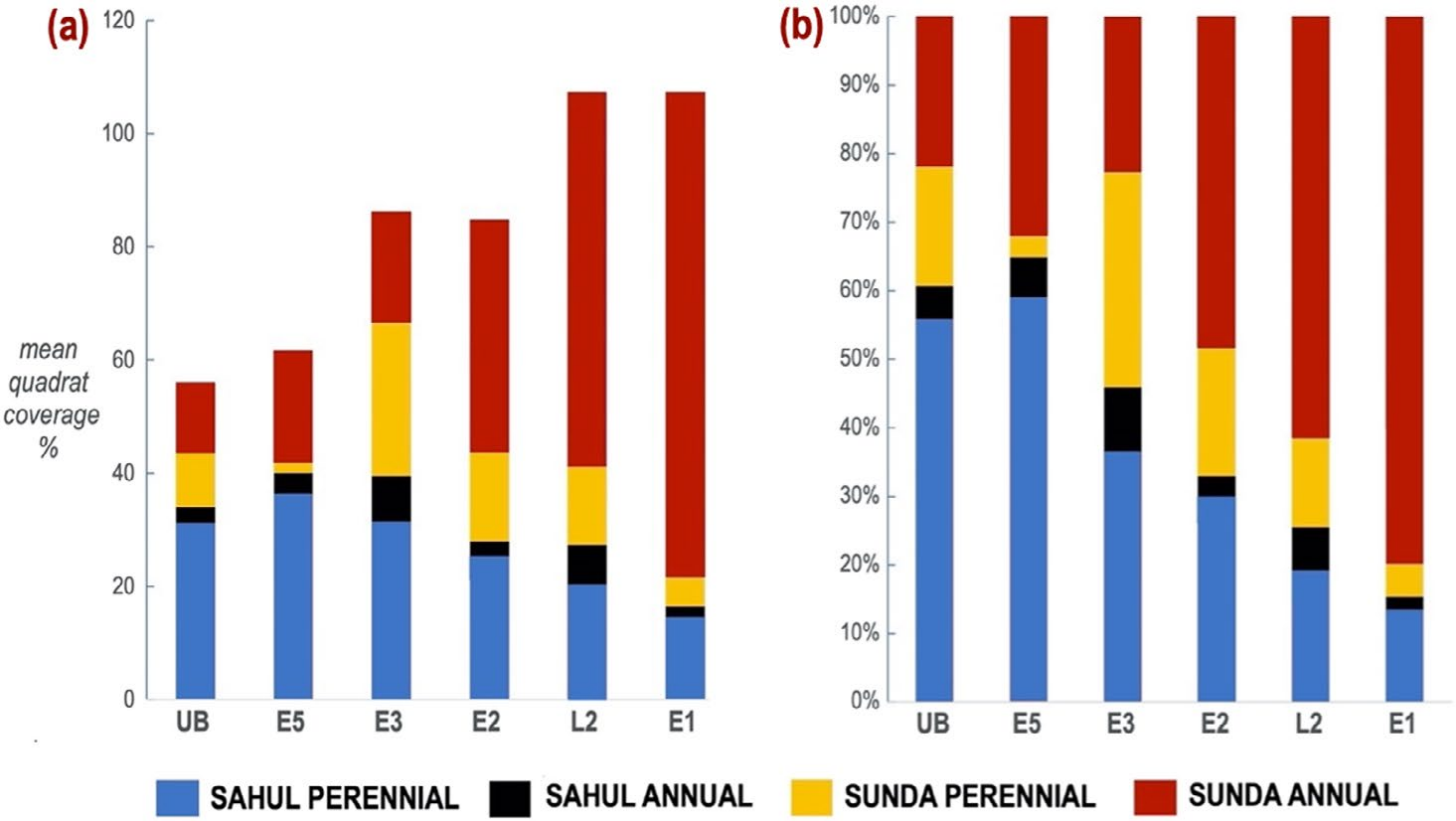
Change in grass types across plots with increasing fire frequency. (a) Abundance of grass species according to evolutionary origins and plant longevity. Note that totals can exceed 100% because of overlapping species in a quadrat. (b) Composition (% of mean total abundance) of grasses across plots with different fire frequencies. Fire frequency is designated as Early season (E) or Late (L), followed by numeral indicating years between fires. UB = Unburned.

The shift in dominance between these biogeographical groups was one of the most striking patterns in this study (Figure 4). Increasing fire frequency more than quadrupled Sundanian species as a proportion of plot coverage in E1 and L2 plots compared to UB and E5. By contrast, Sahul‐origin grasses (mean coverage 37.6% (±7.3%) of UB and E5 plots) were also present in more frequently burned plots, but with much lower and more variable plot coverage (22.% (±15.4%)) in the E1 and L2 plots.

The same suite of perennial species (Appendix S1) was present across all treatment types. The most dominant Sundanian species included tall, bulky grasses of the Andropogoneae tribe (*Sarga intrans, S. plumosum, Chrysopogon fallax*, *Heteropogon triticeu*s).

*Sprouts* are indicators of regeneration. Fire frequency of 2–3 years appeared to stimulate Sunda‐origin sprouts, compared to UB‐E5 groups (Figure 2d; ANOVA *F* = 6.45, df = 2, *p* = 0.007). Sprouts in UB‐E5 plots were dominated by Sahulian species (mean abundance 13.0 (±2.2)) compared to Sundanian species (mean 3.9 (±1.8)). This contrasted with the L2‐E1 plots where Sundanian sprouts had increased to the extent that they were co‐dominant, with Sahulian mean abundance of 10.1 (±2.8) compared to Sundanian mean abundance of 11.7 (±3.6) (Appendix S4). Sprouting of Sahul‐origin species was also stimulated by fire, with peaks in *Acacia* abundance in E2 plots (E3‐E2: L2‐E1, ANOVA *F* = 3.035, df = 2, *p* = 0.078).

*Shrubs*. The effect of fire on the structural profile of woody vegetation was apparent in the progressive proportional decrease in the shrub layer from UB to E5 to E3 (Figure 2c). Shrubs dominated UB plots (60% of all woody stems counted) but in E5 plots, they comprised only 42%, and were well below this in the E3, E2, L2 and E1 plots (34%, 31%, 35% and 37%, respectively). The overall trend of decreasing shrub abundance between UB–E5 and L2–E1 plots was driven by declines in Sahul species (ANOVA *F* = 2.362, df = 2, low‐high, *p* = 0.10). However, Sundanian shrubs showed no similar trend and instead, mean abundance increased between UB–E5 and E3–E2 plots (ANOVA *F* = 3.865, df *= 2*, *p* = 0.044). The small peak in Sahul species abundance in the E2 plots is caused by high numbers of *Acacia* species (see Appendix S5).

*Trees* generally comprised a higher proportion of individual stems in UB and E5 plots (6.7 (±0.28 SE)) and 8.4% (±0.47 SE), respectively. With increasing fire frequency, this proportion fell to less than 6.5%, with a low of 3.9% (±3.91 SE) in L2 plots. This decline is attributed to reductions in the mean abundance of small trees with fire frequency, from 2.33 (±0.3 SE) trees per plot in UB to 0.87 (±0.2 SE) in L2 (see Appendix S6).

*Tall trees* had the fewest individuals of any of the strata in this study, with total mean abundance of 0.91 trees (±0.2)/100 m^2^, overwhelmingly comprised of ancient‐origin species (Figure 2a), particularly *Eucalyptus*. Trees appeared to be unaffected by fire frequency (ANOVA *F* = 0.896, df = 5, *p* = 0.514), except that lowest abundance is found in the L2 plots, where nearly 70% of plots had no tall trees (Table 1). There were only 28 Sunda‐origin tall trees compared with 493 Sahul‐origin.

**TABLE 1:** Quadrats with zero individuals in vegetation categories according to each fire treatment. Treatments with the highest proportion of zero records for each vegetation type are shown in bold. Fire frequency is designated as Early season (E) or Late (L), followed by a numeral indicating years between fires. UB = Unburned. Quadrats = 100 m^2^.

Vegetation	Quadrats with zero individuals (%)					
	UB	E5	E3	E2	L2	E1
Category	(*n* = 93)	(*n* = 90)	(*n* = 102)	(*n* = 91)	(*n* = 98)	(*n* = 99)
Tall trees	54	57	32	24	**69**	41
Small trees	32	19	14	14	**49**	23
Shrubs	0	0	0	0	47	**68**
Sprouts	0	0	0	0	0	**14**

*Small trees* (Figure 2b) showed evidence that increasing fire frequency reduced abundance, particularly in the L2 plots. Sahulian small trees recorded highest mean abundance (1.8 trees (±0.4)/100 m^2^) in the UB‐E5 plots and lowest in the L2‐E1 plots (0.6 (±0.1) trees/100 m^2^). Increasing fire frequency caused a decline in species richness of Sahul‐origin tree species compared to the consistent presence of Sunda‐origin species (except for L2 sites where the fire regime appears to affect survival of all species types) (Appendix S6). This proportional shift also changed canopy phenology as four of the five most abundant Sundanian small trees/shrubs are dry‐season deciduous (Appendices S5 and S6).

### 3.3 Overall Plant Abundance

**FIGURE 5:**
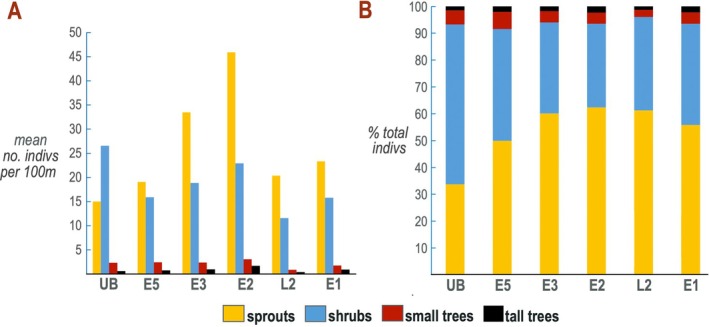
Abundance and composition in woody strata in relation to fire frequency, Berry Springs, Australia. (a): Mean number of individuals per 100 m^2^ (± 1 SD). (b): Proportion of total individuals in each plant category. Fire frequency is designated as Early season (E) or Late (L), followed by numeral indicating years between fires. UB = Unburned.

Tree numbers overall remained few but steady with increasing fire frequency, apart from a marked fall in L2 plots (Figure 5a, Appendix S7). Shrubs were the dominant vegetation form in UB plots but under the E5 regime the dominance shifted to sprouts (Figure 5b), with sprouting plants particularly profuse in the E2 plots (Figure 5a). Differences became more apparent with biogeographical analysis as described above.

While all plots had no trees in some quadrats (Table 1), the only plots that recorded no shrubs were in L2 and E1. The L2 plots can be characterised thus: nearly 70% of plots had no tall trees, 49% had no small trees, and 47% had no shrubs. The impact on shrubs in E1 was even greater, with 68% of plots recording zero shrubs, and an additional 14% of plots had no sprouts.

### 3.4 Leaf Litter, Bare Ground and Shade

There was evidence that fire frequency affected the presence of bare ground (ANOVA *F* = 3.819, df = 5, *p* = 0.0266), covering a mean of 24.3% of L2 plots. There was similar evidence that fire frequency reduced shade in more frequently burned plots than in UB or E5 plots (ANOVA *F* = 2.452, df = 5, *p* = 0.03); see details in Appendix S8. There was evidence of a weak relationship between fire frequency, presence of leaf litter and mean shade coverage in the UB and E5 plots, with leaf litter decreasing by half in the frequently burned plots (ANOVA *F* = 2.452, df = 5, *p* = 0.0943); see details in Appendix S8.

## 4 Discussion

In less than 20 years, increasing fire frequency initiated a system shift from Sahulian to Sundanian vegetation. Nearly every indicator examined found a significant difference between the way modern, Sunda‐origin species responded to increasing fire compared to the effect on biogeographically ancient Australian (Sahulian) species. Dense multi‐strata woodlands dominated by Sahul‐origin species were transformed into tree‐grass savannas with Sundanian species co‐dominant in shrub and sprout layers. This was achieved through a shift in dominance in largely the same suite of species, albeit with the loss of some which appear to be fire‐sensitive.

The clear shift from Sahulian to Sundanian grasses—as fire‐carrying *Sarga* species advanced across the site—was one of the starkest findings of this study, with many of the frequently burned plots covered by a continuous C4 grassy understorey, characteristic of savannas in other countries (Hoffmann et al. 2012). Unusually, however, here they were becoming dominated by annual species rather than perennials. The UB and E5 plots lacked continuous understoreys of flammable C4 grass, but had C4 grasses nonetheless; Sahul‐origin endemic *Eriachne* species provided patchy cover at best. Unusually for a C4 grass, *Eriachne* can grow and flower in shade (Ward and Lane 1986), and their presence throughout the UB and E5 plots suggests a role as co‐habitants in a diverse ecosystem rather than as drivers of change.

Increasingly frequent fire progressively dismantled the Sahul‐origin shrub layer. The reduced abundance of shrubs contrasts with the increased abundance of Sunda‐origin sprouts and portends a shift in future vegetation composition. While Sahul‐origin species also resprouted, the intervals between fires were too short to enable maturation and flowering. These results question the idea that functional traits alone (e.g., resprouting) are enough to enable managers to predict the behaviour and dynamics of plants under different, alternative fire regimes (Keeley et al. 2011).

**FIGURE 6:**
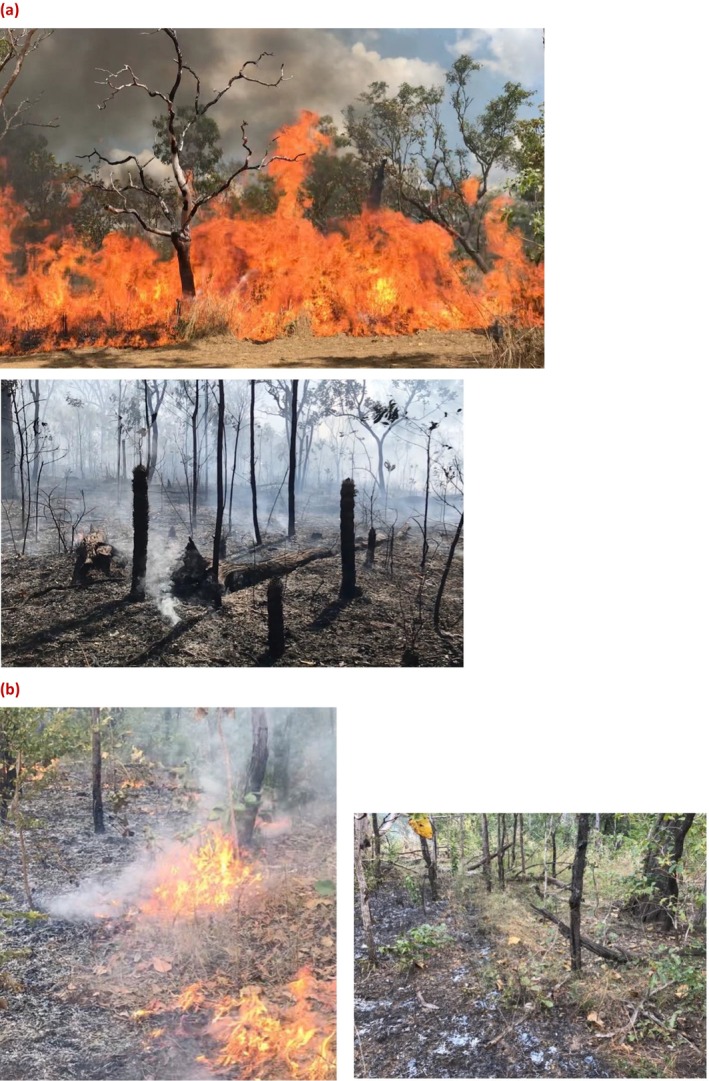
Examples of flame heights and burn coverage in plots ignited early in the dry season at different frequencies. (a) Plot covered in annual *Sarga intrans* and burned yearly or biennially (E1, E2). (b) Plot covered in leaf litter and *Eriachne triseta*, burned at 5‐year frequency (E5).

While fire season is often used as a proxy for severity, heterogeneity in scorch heights has been demonstrated across early and late season fires (Edwards and Russell‐Smith 2009). Our study laid bare the paradox that here, burning every 1–2 years for 20 years, no matter what season, can create the dangerous conditions that fire management aims to prevent, because it creates and sustains conditions for domination by flammable grasses. On E1, L2 and E2 sites that are covered in the annual grass *Sarga intrans*, flame heights of 10 m are typical and sites are > 90% burned (Figure 6a) (Corey et al. 2020). The common assumption that early dry‐season burning benefits biodiversity remains conjecture only, and it may actually be detrimental, especially for taxa that rely on vegetation unburned for longer than 3 years. The other assumption is that EDS burning mirrors indigenous burning practice and creates small patchy mosaics. However, this labour‐intensive attention to fire required cannot be replicated by modern methods of large‐scale EDS burning by dropping incendiaries from helicopters (Corey et al. 2020).

The study also supports others in that while *any* fire changes a dry woodland (Barlow and Peres 2006), frequencies of about 5 years can keep them less permeable to tall flammable grasses (Williams et al. 2003). In our study, flames burn slowly through leaf litter and create patchy burns, akin to traditional indigenous fire practice (Figure 6b).

All the grasses instrumental to the site's transformation belonged to the Andropogoneae tribe, which are inherently more flammable than other grasses (Ripley et al. 2015). The flame heights generated by these taller, larger, more vigorous grasses carry fire higher into shrub layers than occurs with the smaller, finer *Eriachne* (see Figure 2). As Andropogoneae grasses moved into Sahulian ecosystems, the increased height of fire would have been transformative. At this time, the effect on ecosystem structure would have been analogous to the contemporary problem of the introduced and invasive Andropogoneae Gamba grass ( *Andropogon gayanus* ) which carries flames twice as high as *Sarga* (Setterfield et al. 2010), again through vegetation with which it has not co‐evolved.

These changes suggest that the Sunda‐origin shrub and tree species are evolutionarily pre‐adapted to more frequent fires than the Sahulian non‐eucalypt types, and that together with the Andropogoneae grasses, they form a savanna system that has extended its range from Southeast Asia to Australia. As such, we need to think about the development of Australian savannas in a greater cross‐continental context, akin to the way that rainforest research has linked north Queensland, Papua New Guinea, and lands beyond the Wallace Line.

In a broad structural sense, these findings echo savanna studies undertaken across decades in northern Australia (Andersen et al. 2005; Levick et al. 2019; Russell‐Smith et al. 2003; Scott et al. 2012; P. R. Williams et al. 2003) and abroad (Archibald 2016; Ratnam et al. 2011; Sankaran et al. 2005). With increasing fire frequency, the lower strata transformed from fine low grasses and leaf litter to tall bulky grasses and bare ground, and from shrub dominance to sprout dominance. Plant species changed in maturity from small trees to shrub size, to sprouts, and with annual fire, many were absent altogether. This change was evident even at 5‐year fire frequency, with primacy of shrubs being replaced by sprout dominance, a clear example of plants becoming stuck in the ‘fire trap’ and unable to grow clear of the fire danger zone (Bond and Midgley 2001) (see also Appendix S7). In line with other studies, frequent fire here also affected persistence of species which appear to be sensitive to fire (Fensham et al. 2003; Sankaran et al. 2005).

Structural disparities widened with increased fire frequency. Trees that were formerly dry forest emergent were all that remained as everything underneath was burned, to the extent that the L2 and E1 plots could be characterised as ‘open savanna country’, i.e., a system dominated by scattered trees and with an understorey of flammable C_4_ grass (Scott et al. 2012). Structural simplification was highlighted by nearly 50% of L2 plots and nearly 70% of E1 plots having no shrubs, showing how even fire‐tolerant plant species can be overcome by very frequent fire, or fire occurring in a time of plant stress (Bradshaw et al. 2011).

The availability of light is central to understanding the function of tree‐grass ecosystems (Bond 2021; Pilon et al. 2021). Compared to full‐sun situations, grass cover is halved under tree canopies that exceed a Leaf Area Index (LAI) of 1.0 (Pilon et al. 2021) and, with LAI > 1.5, conditions for grass growth become unfavourable. In Australian savannas and woodlands, the dominance of eucalypts (Bowman et al. 2010; P. R. Williams et al. 2003) is a unique difference compared to the light systems of savanna ecosystems elsewhere. The eucalypts in this study (*E. tetradonta, E. miniata*) are common across northern Australia, and are taller and narrower than dominant African savanna trees (e.g., *Vachellia, Senegalia*) (Moncrieff et al. 2014). These eucalypts have the lowest LAI (0.88) of Australia's eucalypts which, in turn, have the lowest LAI of all the world's trees. By contrast, American savanna oaks and pines rate much higher: *Quercus alba* and *Q. marilandica* LAI 5.*2; Q. ellipsoides* and *Q. macrocarpa* LAI 3.3; and *Pinus ponderosa* LAI 4.4 (Iio et al. 2014). It is therefore questionable whether the high eucalypt crowns can provide adequate shade to exclude grass growth once the shrub layer is removed.

Frequent fire also affected openness by increasing the proportions of deciduous Sunda‐origin trees and shrubs. While some northern eucalypts are semi‐deciduous, the most common Sunda‐origin trees in the northern savannas are deciduous in the dry season (R. J. Williams et al. 1997). Here, although overall tree species richness declined with more frequent fire, the increased proportion of Sundanian deciduous species meant that during the dry season, evergreen shrubs were the last providers of full shade. When these were burned, neither the remaining eucalypts nor deciduous trees could create enough consistent shade, with the ensuing high light conditions accelerating the transition to Sundanian annual grasses, namely *Sarga intrans*.

The Sahul‐origin species that remain in the northern savannas today probably evolved in quite different environmental conditions. These are exemplified by fire refuges in the Arnhem sandstone plateau which have the highest plant diversity in the north of the Northern Territory and are more floristically akin to Sahul‐origin‐dominant, southern Australian heathlands than to their neighbouring lowland savannas (Woinarski et al. 2006). Yates et al. (2008) surveyed the extent and frequency of fires in the north of the Northern Territory and found multiple fire‐vulnerable habitats embedded in the broader savanna matrix, including those associated with Sahul‐origin obligate‐seeding plants and marsupials. The low plant endemism of the savannas (Woinarski et al. 2006) reflects their recent advent (Bryceson et al. 2023), with changes in this region most likely being ‘adaptive’ rather than evolutionary, echoing Linder's conclusion that the ‘highly flammable C_4_ grasses may have driven an orgy of extinction’ (Linder 2014).

The responses of vegetation to fire also raise questions about the role of people in shaping regional savannas. Recent fires in Hawai‘i illustrate how invasive C_4_ grasses can drive grass–fire cycles across large areas (Romero and Kovaleski 2023), a process recognised for decades (D'Antonio and Vitousek 1992). Although the antiquity of Australian savannas remains uncertain, the rapid conversion of the TWP dry forest to savanna observed in this study, together with the Hawaiian example, supports suggestions that northern Australian savannas may be relatively young (Bird et al. 2013, 201; Johnson 2016; Van Der Kaars et al. 2000).

Precisely how young is arguable but we posit three key time‐period stages: first, from around 1 Ma (Bryceson and Morgan 2022), highly flammable Andropogoneae grasses—initially sparse in a more shaded landscape with *Eriachne* groundcover—may have dispersed sporadically as opportunities arose after late dry‐season lightning fires. Second, from ~65 ka, Aboriginal burning patterns applied over tens of thousands of years (Johnson 2016, 20), across seasons and guided by cues different from those driving lightning strike, probably promoted open vegetation and grass–fire feedbacks (Bliege Bird et al. 2016). Third, in the past 200 years, European settlers depopulated Aboriginal communities across northern Australia, created a thriving cattle industry, introduced new grasses and replaced Aboriginal burning practices, greatly increasing fire frequency across northern Australia (Crowley and Garnett 1998; Lynch et al. 2007; Woinarski and Legge 2013) (see also Appendix S9).

While some parts of the northern savannas could be hundreds of thousands of years old, it is also possible that extensive areas reflect recent fire regimes greatly exceeding natural return intervals (Brook and Bowman 2006) or documented Indigenous burning patterns (Mooney et al. 2011). The savannas are reputed to be highly fire‐prone (Murphy et al. 2013, 2014; Russell‐Smith et al. 2007; Yates et al. 2008), despite the fact that humans ignite nearly all of the fires (Kelly et al. 2020; Russell‐Smith et al. 2007). As such, these landscapes do not ‘attract’ fire per se, but rather, it is imposed on them and, because of the dominance of Sundanian Andropogoneae grasses, they burn readily. For example, from 1980 to 1994, an average of 46% of Kakadu National Park was burned annually (Russell‐Smith et al. 2007), and from 1999 to 2003, at least 43% of western Cape York burned each year, reaching 74% in 1 year (Felderhof and Gillieson 2006). As Russell‐Smith et al. (2012) warn, the apparently “healthy” savanna landscape may conceal gradual biodiversity loss under intensified fire regimes, potentially reflecting the contraction of formerly more extensive dry forests. The current perception and treatment of this region as ‘ancient savanna’ could be a case of shifting baselines, where contemporary physiognomies are assumed to represent what was ‘always there’.

### 4.1 Contemporary Land Management in Australia

This study supports previous findings that the characterisation of early dry season (EDS) fires as mild and late dry season (LDS) fires as severe is an oversimplification, because fire behaviour and intensity are not strictly constrained by timing within the dry season (Corey et al. 2020) (Figure 6). In northern Australia, management strategies increasingly promote early dry‐season burning to reduce the extent and intensity of late dry‐season fires and associated greenhouse gas emissions (Bowman et al. 2020). In many indigenous communities, carbon‐credit programs aim to provide economic opportunities (Arnhem Land Fire Abatement 2026), the benefits for biodiversity are often assumed rather than demonstrated (Bowman and Legge 2016; Corey et al. 2020). For example, some EDS burning programs have shifted fire timing without reducing the total area burned (Bowman et al. 2020), Additionally, carbon‐credit schemes are funded according to annual fire patterns rather than the long‐term patterning necessary for species requiring long‐unburnt habitat (Clarke et al. 2015). From the perspective of this study, an annual focus shifts the balance against Sahul‐origin plant species in favour of Sundanian elements, with the associated habitat implications.

Unease with savanna fire management regimes is not new, and the need for strategic protection of long‐unburned areas from fire is documented in nearly every savanna study in northern Australia (Clarke et al. 2015). Recent work by Davies et al. (2023) links the application of patchy, regular, early‐season fire with declines in marsupial populations, bringing the efficacy of this widely promoted management regime into question. From an ecological perspective, the habitats supporting Australia's ancient endemic taxa may warrant particular conservation attention because many of these lineages occur nowhere else in the world. Disturbances such as altered fire regimes can affect both the persistence of these fauna and the distinctive vegetation communities with which they co‐evolved.

Mean fire frequency in the northern regions (rainfall > 1000 mm) indicates that much of the land is burned every 1–2 years, and alarmingly, the classification of ‘longer‐unburned’ can have a threshold as low as 3 years (Evans and Russell‐Smith 2020). We show that this frequency will sustain Sunda‐origin annual flammable Andropogoneae grasses at the expense of other forms and this has critical ramifications for the effectiveness of carbon emission reduction programs, given the high intensity of burns they generate.

While, in a structural sense, our findings support savanna studies elsewhere, the lens of biogeographic origin enabled the deeper trends in ecosystem change to become apparent. Applying a biogeographic perspective wherever modern and ancient elements cohabit could help to reduce some of the uncertainty about ecosystem functioning and provide clearer direction for policy.

## Author Contributions

**Susanna Rozsa Bryceson:** conceptualization (equal), data curation (lead), formal analysis (lead), funding acquisition (equal), methodology (equal), project administration (lead), writing – original draft (lead), writing – review and editing (lead). **John William Morgan:** conceptualization (equal), formal analysis (supporting), funding acquisition (equal), methodology (equal), resources (lead), supervision (lead), writing – original draft (supporting), writing – review and editing (supporting).

## Funding

This work was supported by Wiley/Ecological Society of Australia, Fundamental Ecology Award. La Trobe University, Australian Post‐graduate scholarship.

## Conflicts of Interest

The authors declare no conflicts of interest.

## Supporting information


**Appendix S1:** Species list.
**Appendix S2:** Quadrats surveyed.
**Appendix S3:** ANOVA summaries & detail.
**Appendix S4:** Sprouts.
**Appendix S5:** Shrubs.
**Appendix S6:** Small trees.
**Appendix S7:** Summary of mean abundance across the whole site.
**Appendix S8:** Bare ground, leaf litter and shade.
**Appendix S9:** Relationship between lightning and fire in northern Australia.

## Data Availability

https://figshare.com/s/40f750b2a7d56452f2aa.
