# Supplementary material for: Origins Under the Blowtorch: Frequent Fire Shifts the Balance Between Sunda‐Origin and Sahul‐Origin Plant Species in a Tropical Savanna
Source: Ecol Evol. 2026 Jun 21;16(6):e73837. doi: 10.1002/ece3.73837 (PMC13284095; doi:10.1002/ece3.73837)

## APPENDIX: Supporting Information

## **Origins under the blowtorch: frequent fire shifts the balance between Sunda-origin and Sahul-origin plant species in a tropical savanna**

**Susanna Rosza Bryceson & John William Morgan**

Corresponding author: s.bryceson@latrobe.edu.au

**Susanna Rozsa Bryceson** - corresponding author s.bryceson@latrobe.edu.au
Department of Ecological, Plant & Animal Sciences, La Trobe University, Bundoora VIC 3086, AUSTRALIA.

ORCID - 0000-0002-3433-9235

**John William Morgan** - j.morgan@latrobe.edu.au
Department of Ecological, Plant & Animal Sciences, La Trobe University, Bundoora VIC 3086, AUSTRALIA.
ORCID - 0000-0003-2003-5983

JUNE 2026

##

## TABLE OF CONTENTS

### Appendix S1. SPECIES LISTS

Table S1a: Species list: grass genera according to region origin.

Table S1b: Species list: trees and shrubs according to region origin.

### Appendix S2. QUADRATS SURVEYED

Table S2: Numbers of quadrats surveyed in each site and replicate group

### Appendix S3. ANOVA SUMMARIES & DETAIL

Table S3a: Plant category x Treatment effect one-way ANOVA analyses

Table S3b: ANOVA details: plant category x treatment

Table S3c: Summary Mean abundance, all vegetation strata, Block effect. One-way ANOVA analyses

Table S3d: Grass coverage, treatment, origin and life strategy

Table S3e: Block effect on grass coverage

Table S3f: Biogeographical group–life strategy-treatment

### Appendix S4. SPROUTS

Table S4a Sprouts: Mean abundance for each species, in each treatment.

Figure S4b: Shift in mean sprout abundance according to fire frequency.

### Appendix S5. SHRUBS

Figure S5a: Top 5 most abundant shrubs in each origin type.

Figure S5b: Deciduous trees and shrubs in relation to fire frequency.

### Appendix S6. SMALL TREES

Figure S6a: Species richness in small trees by origin throughout fire-frequency treatments.

Figure S6b: Small tree abundance, species composition and distribution of main genera in
 relation to fire frequency, and species origin.

### Appendix S7. SUMMARY OF MEAN ABUNDANCE ACROSS THE WHOLE SITE

Table S7: Abundance and proportion of stems in each non-grass stratum.

### Appendix S8. BARE GROUND, LEAF LITTER AND SHADE

Figure S8: Effect of fire frequency on bare ground and leaf litter, and shade.

### Appendix S9: RELATIONSHIP BETWEEN LIGHTNING AND FIRE IN NORTHERN AUSTRALIA

Figure S9: Comparison of mean monthly distribution of lightning incidence and fire-affected areas across northern Australian regions.

## Appendix S1 Species lists

Species recorded in the vegetation survey. Note that surveys were undertaken during the early dry season. The survey was not intended to be fully comprehensive and only included species more than once, and were of a size to be noticeable when walking in the landscape. Herbaceous plants, forbs and vines were not included.

Pleistocene land masses = Sunda or Sahul as per Joyce et al 2020. Note in text, Sunda is synonymous with 'modern'; Sahul with 'ancient'.

TABLE S1a. SPECIES LIST: grass genera according to region origin.
Origin 'verdict' as per Bryceson & Morgan, 2022. * = annual species.

| **Type** | **Verdict** | **Subfamily / SuperTribe (ST)** | **Tribe** | **Subtribe** | **Genus** | **Species** |
| --- | --- | --- | --- | --- | --- | --- |
| grass | Sahul | Micrairoideae | Micrairoideae | Eriachninae | *Eriachne* | *agrostidea** |
| grass | Sahul | Micrairoideae | Micrairoideae | Eriachninae | *Eriachne* | *avenacea* |
| grass | Sahul | Micrairoideae | Micrairoideae | Eriachninae | *Eriachne* | *burkittii* |
| grass | Sahul | Micrairoideae | Micrairoideae | Eriachninae | *Eriachne* | *ciliata** |
| grass | Sahul | Micrairoideae | Micrairoideae | Eriachninae | *Eriachne* | *stipacea* |
| grass | Sahul | Micrairoideae | Micrairoideae | Eriachninae | *Eriachne* | *triseta* |
| grass | Sahul | Chloridoideae | Cynodonteae | Triodiinae | *Triodia* | *bitextura* |
| grass | non-Sahul | Aristidoideae |  |  | *Aristida* | *holathera** |
| grass | Sunda | Chloridoideae | Eragrostidae | Eragrostidinae | *Eragrostis* | *tenuifolia* |
| grass | Sunda | Chloridoideae | Eragrostidae | Eragrostidinae | *Eragrostis* | *cummingi* |
| grass | Sunda | Panicoideae | Paniceae | Boivinellinae | *Alloteropsis* | *semialata* |
| grass | Sunda | Panicoideae | Paniceae | Panicinae | *Panicum* | *mindanaense* |
| grass | Sunda | Andropogonodae ST | Andropogoneae | Saccharinae | *Pseudopogonatherum* | *contortum* |
| grass | Sunda | Andropogonodae ST | Andropogoneae | Saccharinae | *Sarga* | *plumosum* |
| grass | Sunda | Andropogonodae ST | Andropogoneae | Saccharinae | *Sarga* | *intrans** |
| grass | Sunda | Andropogonodae ST | Andropogoneae | uncertain | *Chrysopogon* | *fallax* |
| grass | Sunda | Andropogonodae ST | Andropogoneae | uncertain | *Chrysopogon* | *latifolius* |
| grass | Sunda | Andropogonodae ST | Andropogoneae | Andropogoninae | *Schizachyrium* | *fragile* |
| grass | Sunda | Andropogonodae ST | Andropogoneae | Andropogoninae | *Themeda* | *triandra* |
| grass | Sunda | Andropogonodae ST | Andropogoneae | Andropogoninae | *Heteropogon* | *triticeus* |
| grass | Sunda | Andropogonodae ST | Andropogoneae | Rottboelliinae | *Mnesithea* | *formosa** |
| grass | Sunda | Andropogonodae ST | Andropogoneae | Rottboelliinae | *Thaumastochloa* | *major** |

TABLE S1b SPECIES LIST: Trees and shrubs according to region origin

Origin 'verdict' arrived at through consideration of Joyce et al 2020, Yap et al 2018; Kooyman 2014; Sniderman & Jordan; G J Jordan pers. comm. (GJJ), Kew Plants of the World (POTW), and other references as cited. Pleistocene land masses = Sunda or Sahul as per Joyce et al 2020. Note in text, Sunda is synonymous with 'modern'; Sahul with 'ancient'.

| **Type** | **Family** | **Genus** | **Species** | **Verdict** | **Reasons for designation** | **Other references** |
| --- | --- | --- | --- | --- | --- | --- |
| shrub-tree | Fabaceae | *Acacia* |  | **Sahul** | Early Oligocene presence in Australia, Sniderman & Jordan 2011. Phylogenetic connections to south, Mishler et al 2014. Sahul: Yap et al 2018. | Mishler et al 2014 |
| shrub-tree | Rhamnaceae | *Alphitonia* | *excelsa* | **Sahul** | Dated to Olgocene in Australia, Richardson et al 2004. Sahul, Yap et al 2018, GJJ | Richardson JE, D. 2004. |
| shrub-tree | Apocynaceae | *Alstonia* | *actinophylla* | **Sunda** | Sunda origin as per Sniderman & Jordan 2011. Genus distribution Joyce et al 2020. |  |
| shrub-tree | Proteaceae | *Banksia* | *dentata* | **Sahul** | Dated to Paleocene-Eocene: Martin 1994, Sniderman & Jordan 2011, Sauquet et al 2009; Barker et al 2007. Sahul: Yap et al 2018. | Martin in Hill, Sauquet et al. 2009; Barker, N. P. et al. 2007 |
| shrub-tree | Sterculiaceae | *Brachychiton* | *diversifolius* | **Sahul** | Centre of diversity of genus. Dated to Middle Eocene, Australia, Sniderman & Jordan 2011. Yap et al 2018. Genus distribution, Joyce et al 2020. |  |
| shrub-tree | Euphorbiaceae, Phyllanthacaceae | *Bridelia* | *tomentosa* | **Sunda** | Pantropical family, Kathriarachchi, et al (2005). Sunda origin as per Yap et al 2018. Species and genus distribution, Joyce et al 2020. | Kathriarachchi, et al (2005). |
| shrub-tree | Anacardiaceae | *Buchanania* | *obovata* | **Sunda** | Sunda origins as per Yap et al, 2018; GJJ 2019; Sniderman & Jordan 2011. Genus distribution Joyce et al 2020. |  |
| shrub-tree | Myrtaceae | *Calytrix* | *brownii* | **Sahul** | Old Australian genus. These species linked to southern origin, Nge et al 2021. | Nge et al, 2021 |
| shrub-tree | Myrtaceae | *Calytrix* | *exstipulata* | **Sahul** | Old Australian genus. These species linked to southern origin, Nge et al 2021. | Nge et al, 2021 |
| shrub-tree | Burseraceae | *Canarium* | *australianum* | **Sunda** | Sunda origin as per Yap et al 2018, Sniderman & Jordan 2011. incense tree, sister to Anacardiaceae. Origins Mexico, Eocene. Sunda migration path to Australia assumed from distribution in Weeks et al 2005. Genus distribution Joyce et al 2020 | Weeks et al 2005. |
| shrub-tree | Rhizophoraceae | *Carallia* | *brachiata* | **Sunda** | Tropical & subtropical family, Xu et al 2017. Species and genus distribution, Joyce et al 2020. GJJ | Xu et al 2017 |
| shrub-tree | Verbenaceae | *Clerodendrum* | *floribundum* | **Sunda** | Tropics and subtropics, Kew POTW. Genus distribution, Joyce et al 2020. GJJ |  |
| shrub-tree | Bixaceae | *Cochlospermum* | *fraseri* | **Sunda** | Sunda origin as per genus distribution, Kew POTW. |  |
| shrub-tree | Rubiaceae | *Coelospermum* | *reticulatum* | **Sunda** | Pantropical family. Genus distribution, Joyce et al, 2020. GJJ. |  |
| shrub-tree | Myrtaceae | *Corymbia* | *bleeseri* | **Sahul** | Palaeocene presence in Australia, Sniderman & Jordan 2011, Thornhill et al 2015. Sahul: Yap et al 2018. | Thornhill et al 2015 |
| shrub-tree | Cycadaceae | *Cycas* | *armstongii* | **Sunda** | Condamine et al 2015 - Cycas are tropical group. Genus distribution, Joyce et al 2020. GJJ | Condamine FL, 2015. |
| shrub-tree | Celastraceae | *Denhamia* | *obscura* | **Sahul** | This is an early diverging lineage in the family, Simmonds et al 2008. Regional distribution as per Sniderman & Jordan 2011, Sunda origin as per Yap et al 2018, and diistribution Australia, NewCal, Kew POTW. Genus distribution Joyce et al 2020. GJJ 2019. |  |
| **Type** | **Family** | **Genus** | **Species** | **Verdict** | **Reasons for designation** | **Other references** |
| shrub-tree | Fabaceae | *Erythrina* | *variegata var orientalis* | **Sunda** | Pan-tropical distribution, Bruneau 1996. Species and genus distribution, Joyce et al 2020. | Bruneau, 1996 |
| shrub-tree | Fabaceae | *Erythrophleum* | *chlorostachys* | **Sunda** | Pan-tropical genus, Pleistocene diversification, Gorel et al 2019. Genus distribution, Joyce et al 2020. | Gorel 2019 |
| shrub-tree | Myrtaceae | *Eucalyptus* | *miniata* | **Sahul** | Palaeocene presence in Australia, Sniderman & Jordan 2011, Thornhill et al 2015. Sahul: Yap et al 2018. | Thornhill et al 2015 |
| shrub-tree | Myrtaceae | *Eucalyptus* | *tetradonta* | **Sahul** | Palaeocene presence in Australia, Sniderman & Jordan 2011, Thornhill et al 2015. Sahul: Yap et al 2018. | Thornhill et al 2015 |
| shrub-tree | Santalaceae | *Exocarpos* | *latifolius* | **Sahul** | Late Oligocene-Early Miocene in Australia, Sniderman & Jordan 2011. Basal stem parasite, Der & Nickrent 2008, Vidal-Russell & Nickrent 2008. GJJ | Vidal-Russell R, Nickrent DL. 2008. Der & Nickrent 2008. |
| shrub-tree | Moraceae | *Ficus* | *aculeata* | **Sunda** | Sunda origin as per Zerega et al 2005, Yap et al 2018. Pan-tropical genus distribution, Kew POTW. | Zerega et al 2005 |
| shrub-tree | Moraceae | *Ficus* | *opposita* | **Sunda** | Sunda origin as per Zerega et al 2005, Yap et al 2018. Pan-tropical genus distribution, Kew POTW. | Zerega et al 2006 |
| shrub-tree | Rubiaceae | *Gardenia* | *megasperma* | **Sunda** | Early diverging tribe from pantropical family, centred In paleotropics, Antonelli et al 2009. Yap et al 2018. Genus distribution, Joyce et al 2020. | Antonelli et al 2009 |
| shrub-tree | Euphorbiaceae / Phyllanthacaceae | *Glochidion* | *xerocarpum* | **Sunda** | Pantropical family, Kathriarachchi, et al (2005). Sunda origin as per Yap et al 2018. Genus distribution, Joyce et al 2020. | Kathriarachchi, et al (2005). |
| shrub-tree | Proteaceae | *Grevillea* | *dryandrii* | **Sahul** | Dated to late Eocene-Oligocene in Australia: Barker et al 2007, Sniderman & Jordan, 2011, Sauquet et al 2009. Sahul, Yap et al 2018. | Sauquet et al. 2009 |
| shrub-tree | Proteaceae | *Grevillea* | *goodii* | **Sahul** | Dated to late Eocene-Oligocene in Australia: Barker et al 2007, Sniderman & Jordan, 2011, Sauquet et al 2009. Sahul, Yap et al 2018. | Sauquet et al. 2009 |
| shrub-tree | Proteaceae | *Grevillea* | *loingicuspis* | **Sahul** | Dated to late Eocene-Oligocene in Australia: Barker et al 2007, Sniderman & Jordan, 2011, Sauquet et al 2009. Sahul, Yap et al 2018. | Sauquet et al. 2009 |
| shrub-tree | Proteaceae | *Grevillea* | *pteridifolia* | **Sahul** | Dated to late Eocene-Oligocene in Australia: Barker et al 2007, Sniderman & Jordan, 2011, Sauquet et al 2009. Sahul, Yap et al 2018. | Sauquet et al. 2009 |
| shrub-tree | Dilleniaceae | *Hibbertia* | *dilatatum* | **Sunda** | Hibbertia is newest clade of sub-tropical / tropical family Dillenaceae, Horn 2009. Regional distribution & recent Sunda origin, Sniderman & Jordan 2011. Distribution based on family. | Horn 2009 |
| shrub-tree | Rubiaceae | *Ixora* | *timorensis* | **Sunda** | Early diverging tribe from pantropical family, centred In paleotropics, Antonelli et al 2009. Yap et al 2018. Species and genus distribution, Joyce et al 2020. | Antonelli et al 2009 |
| shrub-tree | Lauraceae | *Litsea* | *glutinosa* | **Sunda** | Sunda origin as per Yap et al 2018; species and genus distribution, Joyce et al 2020. |  |
| palm | Arecaceae | *Livistona* | *humilis* | **Sunda** | Origin outside Aust & recent immigration, Crisp et al 2010. Genus distribution, Joyce et al 2020. GJJ | Crisp et al 2010 |
| shrub-tree | Celastraceae | *Maytenus* | *ferdinandi* | **Sahul** | This is an early diverging lineage in the family, Simmonds et al 2008. Regional distribution as per Sniderman & Jordan 2011. Sunda origin as per Yap et al 2018. GJJ. Note some uncertainty about Maytenus as monophyletic, with some spp possibly undifferentiated from Denhamia. |  |
| shrub-tree | Myrtaceae | *Melaleuca* | *viridiflora* | **Sahul** | Dated to Eocene, Australia, Thornhill et al 2015. Sahul: Yap et al 2018. | Thornhill et al 2015 |
| shrub-tree | Meliaceae | *Owenia* | *vernicosa* | **Sahul** | Martin, 1994. Old Australian endemic: Koenen et al 2015, GJJ. | Koenen EJM et al 2015. says pan tropical |
| shrub-tree | Pandanaceae | *Pandanus* | *spiralis* | **Sunda** | Tropical, subtropica , Rudall & Bateman 2006, Africa, Asia, Australia, Kew POTW.Genus distribution, Joyce et al 2020. | Rudall & Bateman, 2006. |
| shrub-tree | Proteaceae | *Persoonia* | *falcata* | **Sahul** | Dated to late Eocene-Oligocene in Australia: Barker et al 2007, Sniderman & Jordan, 2011. Sahul, Yap et al 2018. | Sauquet et al. 2009 |
| shrub-tree | Picrodendraceae | *Petalostigma* | *pubescens* | **Sahul** | Paleocene origin, Snderman & Jordan, 2011. Genus and species distribution Joyce et al 2018. |  |
| shrub-tree | Picrodendraceae | *Petalostigma* | *quadriloculare* | **Sahul** | Paleocene origin, Snderman & Jordan, 2011. Genus and species distribution Joyce et al 2018. |  |
| shrub-tree | Lecythidaceae | *Planchonia* | *careya* | **Sunda** | Sunda origin as per Yap et al 2018, genus distribution, Joyce et al 2020. | RK in Yap et al 2018: tropical family |
| tree | Proteaceae | *Stenocarpus* | *acacioides* | **Sunda** | Sniderman & Jordan, 2011: 28.4 Ma, early Oligocene |  |
| shrub-tree | Loganiaceae | *Strychnos* | *lucida* | **Sunda** | Genus pantropical, and temperate South America, Africa, Australia, Kew POTW. Species and Genus distribution, Joyce et al 2020. |  |
| shrub-tree | Myrtaceae | *Syzygium* | *eucalyptoides ssp. bleeseri* | **Sahul** | Dated to Paleocene, Australia, Thornhill et al 2015. Sahul: Yap et al 2018. | Thornhill et al 2015 |
| shrub-tree | Myrtaceae | *Syzygium* | *suborbiculare* | **Sahul** | Dated to Paleocene, Australia, Thornhill et al 2015. Sahul: Yap et al 2018. | Thornhill et al 2015 |
| shrub-tree | Combretaceae | *Terminalia* | *ferdinandiana* | **Sunda** | Regional distribution, Sniderman & Jordan 2011. Tropical family, Sunda origin as per Yap et al 2015. Genus distribution, Joyce et al 2020. | RK in Yap et al 2018: tropical family |
| shrub-tree | Verbenaceae | *Vitex* | *glabrata* | **Sunda** | Tropical / subtropicl to temperate, Kew POTW. Species and genus distribution, Joyce et al. GJJ |  |
| shrub-tree | Myrtaceae | *Xanthostemon* | *paradoxus* | **Sahul** | Dated to Paleocene, Australia, Thornhill et al 2015. Sahul: Yap et al 2018. | Thornhill et al 2015 |

## Appendix S2 Quadrats surveyed

TABLE S2: Numbers of quadrats surveyed in each site and replicate group

Treatment: E=Early season, L=Late season. Numerals = years between fires. UB=Unburned. Site = replicate ID.

| **Treatment** | **Plot** | ***n* 100 m^2^ quadrats** | **total quadrats / treatment** |
| --- | --- | --- | --- |
| E1 | A3 | 34 |  |
| E1 | B2 | 30 |  |
| E1 | D1 | 35 | E1=99 |
| L2 | A2 | 34 |  |
| L2 | B3 | 32 |  |
| L2 | C2 | 32 | L2=98 |
| E2 | A5 | 32 |  |
| E2 | B5 | 27 |  |
| E2 | C6 | 32 | E2=91 |
| E3 | A1 | 36 |  |
| E3 | B4 | 32 |  |
| E3 | C1 | 34 | E3=102 |
| E5 | A4 | 30 |  |
| E5 | B1 | 30 |  |
| E5 | C4 | 30 | E5=90 |
| UB | A6 | 31 |  |
| UB | B6 | 28 |  |
| UB | C5 | 34 | UB=93 |
|  |  |  | **TOTAL 573** |
|  |  |  |  |

## Appendix S3: ANOVA Summaries and detail

TABLE S3a: Plant category x Treatment effect one-way ANOVA analyses
6 treatments = UB, E5, E3, E2, L2, E1.

|  | n treatments | all | ancient | modern (p) |
| --- | --- | --- | --- | --- |
| Sprouts | 6 | 0.062  a=UB  ab= E1, E3, E5, L2  b=E2 | NS | 0.0689  a=E2  ab=E3, E5, UB  b=L2, E1 |
| Shrubs | 6 | NS | NS | NS |
| Small trees | 6 | NS | NS | 0.05  E2 = a  ab = E2, E1  b = UB, E5, L2 |
| Tall trees | 6 | 0.00772**  a= E2,  ab= E3,  b= L2, E1, UB | NS | [note: very small sample size]  0.00224**  a=E2  b=E1, E3, E5, UB, L2 |
| Grass | 6 | NS | NS | 0.00635**  a= E1, L2  ab=E3 E2  b=UB, E5 |
| Bare ground | 6 | 0.0266*  a=L2  ab=E1, E5, UB  b = EE, E3 |  |  |
| Leaf litter | 6 | 0.0943  a=E5  b=L2  ab=E1, E2, E3, UB |  |  |
| Shade | 6 | 0.03*  a=UB  b=L2  ab=E1, E2, E3, E5 |  |  |

| TABLE S3b: ANOVA details: Plant category x Treatment effect | |  | |
| --- | --- | --- | --- |
| ANOVA RESULTS | TUKEY HSD (significant results only) | |  |
| GRASS | GRASS | |  |
| ANCIENT GRASS X TREATMENT | TukeyHSD MODERN GRASS X TREATMENT | |  |
| Df Sum Sq Mean Sq F value Pr(>F) | Tukey multiple comparisons of means | |  |
| Treat 5 1 200 240.0 0.957 0.481 | 95% family-wise confidence level | |  |
| Residuals 12 3010 250.8 | diff lwr upr p adj | |  |
|  | E2-E1 -33.250000 -91.6523232 25.152323 0.4398614 | |  |
| MODERN GRASS X TREATMENT | E3-E1 -44.366667 -102.7689899 14.035657 0.1836397 | |  |
| Df Sum Sq Mean Sq F value Pr(>F) | E5-E1 -69.256667 -127.6589899 -10.854343 0.0174436 | |  |
| Treat 5 12952 2590.4 5.712 0.00635 ** | L2-E1 -11.500000 -69.9023232 46.902323 0.9830987 | |  |
| Residuals 12 5442 453.5 | UB-E1 -71.640000 -130.0423232 -13.237677 0.0138451 | |  |
| Signif. codes: 0 ‘***’ 0.001 ‘**’ 0.01 ‘*’ 0.05 ‘.’ 0.1 ‘ ’ 1 | E3-E2 -11.116667 -69.5189899 47.285657 0.9854343 | |  |
|  | E5-E2 -36.006667 -94.4089899 22.395657 0.3616012 | |  |
| ALL GRASS X TREATMENT | L2-E2 21.750000 -36.6523232 80.152323 0.8045396 | |  |
| Df Sum Sq Mean Sq F value Pr(>F) | UB-E2 -38.390000 -96.7923232 20.012323 0.3015314 | |  |
| TreatAll 5 7018 1403.5 2.302 0.11 | E5-E3 -24.890000 -83.2923232 33.512323 0.7093238 | |  |
| Residuals 12 7317 609.7 | L2-E3 32.866667 -25.5356565 91.268990 0.4514086 | |  |
|  | UB-E3 -27.273333 -85.6756565 31.128990 0.6315039 | |  |
| SHRUBS | L2-E5 57.756667 -0.6456565 116.158990 0.0532049 | |  |
| ANCIENT SHRUBS X TREATMENT | UB-E5 -2.383333 -60.7856565 56.018990 0.9999911 | |  |
| Df Sum Sq Mean Sq F value Pr(>F) | UB-L2 -60.140000 -118.5423232 -1.737677 0.0422791 | |  |
| TreatSh 5 419.9 83.98 1.794 0.188 |  | |  |
| Residuals 12 561.6 46.80 |  | |  |
|  | SMALL TREES | |  |
| MODERN SHRUBS X TREATMENT) | TukeyHSD MODERN SMALL TREES X TREATMENT | |  |
| Df Sum Sq Mean Sq F value Pr(>F) | Tukey multiple comparisons of means | |  |
| TreatSh 5 51.07 10.213 1.807 0.186 | 95% family-wise confidence level | |  |
| Residuals 12 67.83 5.653 | diff lwr upr p adj | |  |
|  | E2-E1 0.41666667 -0.7313332 1.56466657 0.8198798 | |  |
| ALL SHRUBS X TREATMENT | E3-E1 0.83000000 -0.3179999 1.97799990 0.2206297 | |  |
| Df Sum Sq Mean Sq F value Pr(>F) | E5-E1 -0.20666667 -1.3546666 0.94133323 0.9886258 | |  |
| TreatAll 5 507.4 1 01.48 1.833 0.181 | L2-E1 -0.37333333 -1.5213332 0.77466657 0.8753679 | |  |
| Residuals 12 664.5 55.38 | UB-E1 -0.26000000 -1.4079999 0.88799990 0.9692404 | |  |
| SMALL TREES | E3-E2 0.41333333 -0.7346666 1.56133323 0.8244794 | |  |
| ANCIENT SMALL TREES X TREATMENT | E5-E2 -0.62333333 -1.7713332 0.52466657 0.4870626 | |  |
| Df Sum Sq Mean Sq F value Pr(>F) | L2-E2 -0.79000000 -1.9379999 0.35799990 0.2611469 | |  |
| TreatSm 5 5.941 1.188 1.181 0.374 | UB-E2 -0.67666667 -1.8246666 0.47133323 0.4054561 | |  |
| Residuals 12 1 2.070 1.006 | E5-E3 -1.03666667 -2.1846666 0.11133323 0.0857817 | |  |
|  | L2-E3 -1.20333333 -2.3513332 -0.05533343 0.0380917 | |  |
| MODERN SMALL TREES X TREATMENT | UB-E3 -1.09000000 -2.2379999 0.05799990 0.0663439 | |  |
| Df Sum Sq Mean Sq F value Pr(>F) | L2-E5 -0.16666667 -1.3146666 0.98133323 0.9957343 | |  |
| TreatSm 5 3.254 0.6508 3.714 0.029 * | UB-E5 -0.05333333 -1.2013332 1.09466657 0.9999831 | |  |
| Residuals 12 2.103 0.1752 | UB-L2 0.11333333 -1.0346666 1.26133323 0.9993165 | |  |
| Signif. codes: 0 ‘***’ 0.001 ‘**’ 0.01 ‘*’ 0.05 ‘.’ 0.1 ‘ ’ 1 | UB-L2 0.23666667 -0.48974896 0.96308229 0.8745673 | |  |
| ALL SMALL TREES X TREATMENT |  | |  |
| Df Sum Sq Mean Sq F value Pr(>F) |  | |  |
| TreatAll 5 8..277 1.655 1.418 0.286 | TALL TREES | |  |
| Residuals 12 14.006 1.167 | | TukeyHSD MODERN TALL TREES X TREATMENT | |
|  | | diff lwr upr p adj | |
|  | | E2-E1 0.79666667 0.07025104 1.52308229 0.0289333 | |
| TALL TREES | | E3-E1 0.03333333 -0.69308229 0.75974896 0.9999841 | |
| ANCIENT TALL TREES X TREATMENT | | E5-E1 -0.06666667 -0.79308229 0.65974896 0.9995200 | |
| Df Sum Sq Mean Sq F value Pr(>F) | | L2-E1 -0.43333333 -1.15974896 0.29308229 0.3936660 | |
| TreatTTr 5 0.02964 0.005929 0.896 0.514 | | UB-E1 -0.19666667 -0.92308229 0.52974896 0.9367113 | |
| Residuals 12 0.07940 0.006617 | | E3-E2 -0.76333333 -1.48974896 -0.03691771 0.0375306 | |
|  | | E5-E2 -0.86333333 -1.58974896 -0.13691771 0.0171855 | |
| MODERN TALL TREES X TREATMENT | | L2-E2 -1.23000000 -1.95641562 -0.50358438 0.0010950 | |
| Df Sum Sq Mean Sq F value Pr(>F) | | UB-E2 -0.99333333 -1.71974896 -0.26691771 0.0062883 | |
| TreatTTr 5 2.5912 0.5182 7.387 0.00224 ** | | E5-E3 -0.10000000 -0.82641562 0.62641562 0.9966706 | |
| Residuals 12 0.8419 0.0702 | | L2-E3 -0.46666667 -1.19308229 0.25974896 0.3226216 | |
|  | | UB-E3 -0.23000000 -0.95641562 0.49641562 0.8865736 | |
| Signif. codes: 0 ‘***’ 0.001 ‘**’ 0.01 ‘*’ 0.05 ‘.’ 0.1 ‘ ’ 1 | | L2-E5 -0.36666667 -1.09308229 0.35974896 0.5587386 | |
|  | | UB-E5 -0.13000000 -0.85641562 0.59641562 0.9889226 | |
| ALL TALL TREES X TREATMENT | | UB-L2 0.23666667 -0.48974896 0.96308229 0.8745673 | |
| Df Sum Sq Mean Sq F value Pr(>F) | |  | |
| TreatAll 5 2.896 0.5792 5 .428 0.00772 ** | | TukeyHSD ALL TALL TREES X TREATMENT | |
| Residuals 12 1.280 0.1067 | | Tukey multiple comparisons of means | |
| Signif. codes: 0 ‘***’ 0.001 ‘**’ 0.01 ‘*’ 0.05 ‘.’ 0.1 ‘ ’ 1 | | 95% family-wise confidence level | |
|  | | diff lwr upr p adj | |
| a= E2 | E2-E1 0.79250246 -0.1033884 1.6883934 0.0948814 | |  |
| b= L2, E1, UB | E3-E1 0.05854731 -0.8373436 0.9544382 0.9999089 | |  |
| ab= E3 | E5-E1 -0.14229692 -1.0381878 0.7535940 0.9935441 | |  |
|  | L2-E1 -0.48870215 -1.3845930 0.4071887 0.4824580 | |  |
|  | UB-E1 -0.27434866 -1.1702396 0.6215422 0.8993935 | |  |
|  | E3-E2 -0.73395516 -1.6298460 0.1619357 0.1348145 | |  |
|  | E5-E2 -0.93479938 -1.8306903 -0.0389085 0.0391337 | |  |
|  | L2-E2 -1.28120461 -2.1770955 -0.3853137 0.0044504 | |  |
|  | UB-E2 -1.06685113 -1.9627420 -0.1709602 0.0169588 | |  |
|  | E5-E3 -0.20084423 -1.0967351 0.6950467 0.9705273 | |  |
|  | L2-E3 -0.54724946 -1.4431403 0.3486414 0.3705477 | |  |
|  | UB-E3 -0.33289597 -1.2287869 0.5629949 0.8059144 | |  |
|  | L2-E5 -0.34640523 -1.2422961 0.5494857 0.7805389 | |  |
|  | UB-E5 -0.13205175 -1.0279426 0.7638391 0.9954237 | |  |
|  | UB-L2 0.21435348 -0.6815374 1.1102444 0.9613820 | |  |
|  |  | |  |
| SPROUTS | SPROUTS | |  |
| ANCIENT SPROUTS X TREATMENT) | TukeyHSD MODERN SPROUTS X TREATMENT | |  |
| Df Sum Sq Mean Sq F value Pr(>F) | diff lwr upr p adj | |  |
| TreatSp 5 678.8 135.75 2.096 0.136 | E2-E1 16.7000000 -2.356023 35.756023 0.0992319 | |  |
| Residuals 12 777.2 64.77 | E3-E1 2.9833333 -16.072690 22.039357 0.9939575 | |  |
|  | E5-E1 4.1500000 -14.906023 23.206023 0.9739209 | |  |
| MODERN SPROUTS X TREATMENT | L2-E1 -1.5233333 -20.579357 17.532690 0.9997550 | |  |
| Df Sum Sq Mean Sq F value Pr(>F) | UB-E1 0.2266667 -18.829357 19.282690 1.0000000 | |  |
| TreatSm 5 668.2 133.64 2.768 0.0689 . | E3-E2 -13.7166667 -32.772690 5.339357 0.2241156 | |  |
| Residuals 12 579.3 48.28 | E5-E2 -12.5500000 -31.606023 6.506023 0.2998127 | |  |
| Signif. codes: 0 ‘***’ 0.001 ‘**’ 0.01 ‘*’ 0.05 ‘.’ 0.1 ‘ ’ 1 | L2-E2 -18.2233333 -37.279357 0.832690 0.0638681 | |  |
|  | UB-E2 -16.4733333 -35.529357 2.582690 0.1058430 | |  |
| a=E2 | E5-E3 1.1666667 -17.889357 20.222690 0.9999339 | |  |
| b= L2 E1 | L2-E3 -4.5066667 -23.562690 14.549357 0.9631853 | |  |
| ab = E3, E5, UB | UB-E3 -2.7566667 -21.812690 16.299357 0.9958047 | |  |
|  | L2-E5 -5.6733333 -24.729357 13.382690 0.9092621 | |  |
| ALL SPROUTS X TREATMENT) | UB-E5 -3.9233333 -22.979357 15.132690 0.9794910 | |  |
| Df Sum Sq Mean Sq F value Pr(>F) | UB-L2 1.7500000 -17.306023 20.806023 0.9995184 | |  |
| TreatAll 5 2050 410.0 2.877 0.062 . |  | |  |
| Residuals 12 1710 142.5 |  | |  |
| Signif. codes: 0 ‘***’ 0.001 ‘**’ 0.01 ‘*’ 0.05 ‘.’ 0.1 ‘ ’ 1 | TukeyHSD ALL SPROUTS X TREATMENT | |  |
|  | Tukey multiple comparisons of means | |  |
| a=UB | 95% family-wise confidence level | |  |
| b=E2 | diff lwr upr p adj | |  |
| ab=E1 E3 E5 L2 | E2-E1 22.5650878 -10.17425 55.304423 0.2598079 | |  |
|  | E3-E1 10.1225724 -22.61676 42.861908 0.8958315 | |  |
|  | E5-E1 -4.2913165 -37.03065 28.448019 0.9973548 | |  |
|  | L2-E1 -4.7224031 -37.46174 28.016932 0.9958608 | |  |
|  | UB-E1 -8.3684031 -41.10774 24.370932 0.9495407 | |  |
|  | E3-E2 -12.4425154 -45.18185 20.296820 0.7918110 | |  |
|  | E5-E2 -26.8564043 -59.59574 5.882931 0.1340581 | |  |
|  | L2-E2 -27.2874909 -60.02683 5.451844 0.1250062 | |  |
|  | UB-E2 -30.9334909 -63.67283 1.805845 0.0680773 | |  |
|  | E5-E3 -14.4138889 -47.15322 18.325446 0.6827790 | |  |
|  | L2-E3 -14.8449755 -47.58431 17.894360 0.6576203 | |  |
|  | UB-E3 -18.4909754 -51.23031 14.248360 0.4478201 | |  |
|  | L2-E5 -0.4310866 -33.17042 32.308249 1.0000000 | |  |
|  | UB-E5 -4.0770865 -36.81642 28.662249 0.9979229 | |  |
|  | UB-L2 -3.6459999 -36.38534 29.093335 0.9987799 | |  |
|  |  | |  |
| LEAF LITTER | LEAF LITTER | |  |
| LEAF LITTER X TREATMENT | TukeyHSD LEAF LITTER X TREATMENT | |  |
| Df Sum Sq Mean Sq F value Pr(>F) | Tukey multiple comparisons of means | |  |
| LSTreat 5 3234 646.8 2.452 0.0943 . | 95% family-wise confidence level | |  |
| Residuals 12 3165 263.8 | diff lwr upr p adj | |  |
| Signif. codes: 0 ‘***’ 0.001 ‘**’ 0.01 ‘*’ 0.05 ‘.’ 0.1 ‘ ’ 1 | E2-E1 9.433333 -35.10987 53.976540 0.9768464 | |  |
|  | E3-E1 3.300000 -41.24321 47.843207 0.9998312 | |  |
|  | E5-E1 33.966667 -10.57654 78.509873 0.1809673 | |  |
|  | L2-E1 -1.266667 -45.80987 43.276540 0.9999985 | |  |
|  | UB-E1 25.900000 -18.64321 70.443207 0.4189203 | |  |
|  | E3-E2 -6.133333 -50.67654 38.409873 0.9966670 | |  |
|  | E5-E2 24.533333 -20.00987 69.076540 0.4728622 | |  |
|  | L2-E2 -10.700000 -55.24321 33.843207 0.9607475 | |  |
|  | UB-E2 16.466667 -28.07654 61.009873 0.8090294 | |  |
|  | E5-E3 30.666667 -13.87654 75.209873 0.2607541 | |  |
|  | L2-E3 -4.566667 -49.10987 39.976540 0.9991799 | |  |
|  | UB-E3 22.600000 -21.94321 67.143207 0.5537526 | |  |
|  | L2-E5 -35.233333 -79.77654 9.309873 0.1563375 | |  |
|  | UB-E5 -8.066667 -52.60987 36.476540 0.9883199 | |  |
|  | UB-L2 27.166667 -17.37654 71.709873 0.3720550 | |  |
|  |  | |  |
| SHADE | SHADE | |  |
| SHADE X TREATMENT | TukeyHSD SHADE X TREATMENT | |  |
| > ano52 <- aov(shade ~ LSTreat, data = LSFrame) | Tukey multiple comparisons of means | |  |
| > summary (ano52) | 95% family-wise confidence level | |  |
| Df Sum Sq Mean Sq F value Pr(>F) | diff lwr upr p adj | |  |
| LSTreat 5 4.277 0.8554 3.664 0.0303 * | E2-E1 0.38195223 -0.9432063 1.7071107 0.9195818 | |  |
| Residuals 12 2.802 0.2335 | E3-E1 0.28398109 -1.0411774 1.6091396 0.9756467 | |  |
| Signif. codes: 0 ‘***’ 0.001 ‘**’ 0.01 ‘*’ 0.05 ‘.’ 0.1 ‘ ’ 1 | E5-E1 0.43426704 -0.8908915 1.7594256 0.8719911 | |  |
|  | L2-E1 -0.57100257 -1.8961611 0.7541559 0.7004909 | |  |
| a=ub | UB-E1 1.04848273 -0.2766758 2.3736412 0.1561583 | |  |
| b=L2 | E3-E2 -0.09797113 -1.4231296 1.2271874 0.9998329 | |  |
| ab=E1,E2,E3,E5 | E5-E2 0.05231481 -1.2728437 1.3774733 0.9999925 | |  |
|  | L2-E2 -0.95295479 -2.2781133 0.3722037 0.2248671 | |  |
|  | UB-E2 0.66653050 -0.6586280 1.9916890 0.5621428 | |  |
|  | E5-E3 0.15028595 -1.1748726 1.4754445 0.9986689 | |  |
|  | L2-E3 -0.85498366 -2.1801422 0.4701748 0.3186288 | |  |
|  | UB-E3 0.76450163 -0.5606569 2.0896601 0.4267024 | |  |
|  | L2-E5 -1.00526961 -2.3304281 0.3198889 0.1846399 | |  |
|  | UB-E5 0.61421569 -0.7109428 1.9393742 0.6382318 | |  |
|  | UB-L2 1.61948529 0.2943268 2.9446438 0.0142064 | |  |
|  |  | |  |
|  |  | |  |
| BARE GROUND | BARE GROUND | |  |
| BAREGROUND X TREATMENT | > TukeyHSD BARE GROUND | |  |
| Fit: aov(formula = BG ~ bgTreat, data = BGFrame) | Tukey multiple comparisons of means | |  |
| > summary (ano61) | 95% family-wise confidence level | |  |
| Df Sum Sq Mean Sq F value Pr(>F) | diff lwr upr p adj | |  |
| bgTreat 5 841.9 168.39 3.819 0.0266 * | E2-E1 -10.1666667 -28.377264 8.043931 0.4593731 | |  |
| Residuals 12 529.1 44.09 | E3-E1 -3.8666667 -22.077264 14.343931 0.9765877 | |  |
| Signif. codes: 0 ‘***’ 0.001 ‘**’ 0.01 ‘*’ 0.05 ‘.’ 0.1 ‘ ’ 1 | E5-E1 -1.4666667 -19.677264 16.743931 0.9997459 | |  |
|  | L2-E1 12.7000000 -5.510597 30.910597 0.2498725 | |  |
| A=L2 | UB-E1 -1.3000000 -19.510597 16.910597 0.9998593 | |  |
| b=E2, E3 | E3-E2 6.3000000 -11.910597 24.510597 0.8460547 | |  |
| ab = E!, E5 UB | E5-E2 8.7000000 -9.510597 26.910597 0.6107333 | |  |
|  | L2-E2 22.8666667 4.656069 41.077264 0.0117533 | |  |
|  | UB-E2 8.8666667 -9.343931 27.077264 0.5930637 | |  |
|  | E5-E3 2.4000000 -15.810597 20.610597 0.9972862 | |  |
|  | L2-E3 16.5666667 -1.643931 34.777264 0.0826767 | |  |
|  | UB-E3 2.5666667 -15.643931 20.777264 0.9962834 | |  |
|  | L2-E5 14.1666667 -4.043931 32.377264 0.1672617 | |  |
|  | UB-E5 0.1666667 -18.043931 18.377264 1.0000000 | |  |
|  | UB-L2 -14.0000000 -32.210597 4.210597 0.1753062 | |  |
|  |  | |  |
| ALL VEGETATION | ALL VEGETATION | |  |
| ALL VEG X TREATMENT | > TukeyHSD ALL VEGETATION | |  |
| summary (anova100) | Tukey multiple comparisons of means | |  |
| Df Sum Sq Mean Sq F value Pr(>F) | 95% family-wise confidence level | |  |
| allTREAT 5 2.904 0.5807 5.386 0.00795 ** | diff lwr upr p adj | |  |
| Residuals 12 1.294 0.1078 | E2-E1 0.7933333 -0.1071944 1.69386107 0.0967783 | |  |
| --- | E3-E1 0.0600000 -0.8405277 0.96052774 0.9998998 | |  |
| Signif. codes: 0 ‘***’ 0.001 ‘**’ 0.01 ‘*’ 0.05 ‘.’ 0.1 ‘ ’ 1 | E5-E1 -0.1433333 -1.0438611 0.75719440 0.9934820 | |  |
|  | L2-E1 -0.4900000 -1.3905277 0.41052774 0.4849550 | |  |
|  | UB-E1 -0.2733333 -1.1738611 0.62719440 0.9025910 | |  |
|  | E3-E2 -0.7333333 -1.6338611 0.16719440 0.1383540 | |  |
|  | E5-E2 -0.9366667 -1.8371944 -0.03613893 0.0398708 | |  |
|  | L2-E2 -1.2833333 -2.1838611 -0.38280560 0.0045742 | |  |
|  | UB-E2 -1.0666667 -1.9671944 -0.16613893 0.0175790 | |  |
|  | E5-E3 -0.2033333 -1.1038611 0.69719440 0.9696294 | |  |
|  | L2-E3 -0.5500000 -1.4505277 0.35052774 0.3706919 | |  |
|  | UB-E3 -0.3333333 -1.2338611 0.56719440 0.8082539 | |  |
|  | L2-E5 -0.3466667 -1.2471944 0.55386107 0.7834587 | |  |
|  | UB-E5 -0.1300000 -1.0305277 0.77052774 0.9958451 | |  |
|  | UB-L2 0.2166667 -0.6838611 1.11719440 0.9604903 | |  |
|  |  | |  |
| ALL ANCIENT VEG X TREATMENT | TukeyHS ALL ANCIENT VEG X TREATMENT | |  |
| Df Sum Sq Mean Sq F value Pr(>F) | Tukey multiple comparisons of means | |  |
| allTREAT 5 2.5912 0.5182 7.387 0.00224 ** | 95% family-wise confidence level | |  |
| Residuals 12 0.8419 0.0702 |  | |  |
| --- | diff lwr upr p adj | |  |
| Signif. codes: 0 ‘***’ 0.001 ‘**’ 0.01 ‘*’ 0.05 ‘.’ 0.1 ‘ ’ 1 | E2-E1 0.79666667 0.07025104 1.52308229 0.0289333 | |  |
|  | E3-E1 0.03333333 -0.69308229 0.75974896 0.9999841 | |  |
|  | E5-E1 -0.06666667 -0.79308229 0.65974896 0.9995200 | |  |
| ALL MODERN VEG X TREATMENT | L2-E1 -0.43333333 -1.15974896 0.29308229 0.3936660 | |  |
| Df Sum Sq Mean Sq F value Pr(>F) | UB-E1 -0.19666667 -0.92308229 0.52974896 0.9367113 | |  |
| allTREAT 5 0.02964 0.005929 0.896 0.514 | E3-E2 -0.76333333 -1.48974896 -0.03691771 0.0375306 | |  |
| Residuals 12 0.07940 0.006617 | E5-E2 -0.86333333 -1.58974896 -0.13691771 0.0171855 | |  |
|  | L2-E2 -1.23000000 -1.95641562 -0.50358438 0.0010950 | |  |
|  | UB-E2 -0.99333333 -1.71974896 -0.26691771 0.0062883 | |  |
|  | E5-E3 -0.10000000 -0.82641562 0.62641562 0.9966706 | |  |
|  | L2-E3 -0.46666667 -1.19308229 0.25974896 0.3226216 | |  |
|  | UB-E3 -0.23000000 -0.95641562 0.49641562 0.8865736 | |  |
|  | L2-E5 -0.36666667 -1.09308229 0.35974896 0.5587386 | |  |
|  | UB-E5 -0.13000000 -0.85641562 0.59641562 0.9889226 | |  |
|  | UB-L2 0.23666667 -0.48974896 0.96308229 0.8745673 | |  |
|  |  | |  |

TABLE S3c: SUMMARY Mean abundance, all vegetation strata, Block effect. One-way ANOVA analyses

| VEGETATION | n blocks | All | Ancient | Modern | Tukey HSD |
| --- | --- | --- | --- | --- | --- |
| Sprouts | 3 | NS | NS | NS |  |
| Grass | 3 | NS | NS | NS |  |
| Shrubs | 3 | NS | NS | NS |  |
| Small trees | 3 | C BLOCK, 0.4, 0.1 | C - B BLOCK, 0.05* | 0.029* | a=E3  ab=E1 E2  b=E5, L2, UB |
| Tall trees | 3 | NS | NS | NS |  |
|  |  |  |  |  |  |
| NON-VEGETATION | | | | | |
| Bare ground | 3 | NS | NS | NS |  |
| Leaf litter | 3 | NS | NS | NS |  |
| Shade | 3 | NS | NS | NS |  |

ANOVA DETAILS BLOCK EFFECTS (FF)

| BLOCK FF - ALL ANCIENT VEG | NS |
| --- | --- |
| Df Sum Sq Mean Sq F value Pr(>F) |  |
| Siteblok 2 0.184 0.09201 0.425 0.662 |  |
| Residuals 15 3.249 0.21660 |  |
|  |  |
| BLOCK FF - ALL MODERN VEG | NS |
| Df Sum Sq Mean Sq F value Pr(>F) |  |
| Siteblok 2 0.01701 0.008506 1.386 0.28 |  |
| Residuals 15 0.09203 0.006136 |  |
|  |  |
| BLOCK FF - ALL VEG | NS |
| Df Sum Sq Mean Sq F value Pr(>F) |  |
| Siteblok 2 0.0734 0.03670 0.564 0.58 |  |
| Residuals 15 0.9759 0.06506 |  |
|  |  |
| BLOCK FF ANCIENT SPROUTS |  |
| Df Sum Sq Mean Sq F value Pr(>F) | NS |
| SprSiteblok 2 324.8 162.40 2.64 0.104 |  |
| Residuals 15 922.7 61.52 |  |
|  |  |
| BLOCK FF MODERN SPROUTS |  |
| Df Sum Sq Mean Sq F value Pr(>F) | NS |
| SprSiteblok 2 165.6 82.78 0.962 0.404 |  |
| Residuals 15 1290.4 86.03 |  |
|  |  |
| BLOCK FF ALL SPROUTS |  |
| Df Sum Sq Mean Sq F value Pr(>F) | NS |
| SprSiteblok 2 193.8 96.92 1.978 0.173 |  |
| Residuals 15 734.9 49.00 |  |
|  |  |
| BLOCK FF - ALL ANCIENT GRASS | NS |
| Df Sum Sq Mean Sq F value Pr(>F) |  |
| SiteGrassblok 2 175 87.45 0.325 0.727 |  |
| Residuals 15 4034 268.96 |  |
|  |  |
| BLOCK FF - ALL MOD GRASS | NS |
| Df Sum Sq Mean Sq F value Pr(>F) |  |
| SiteGrassblok 2 1430 714.8 0.632 0.545 |  |
| Residuals 15 16964 1130.9 |  |
|  |  |
| BLOCK FF - ALL GRASS | NS |
| Df Sum Sq Mean Sq F value Pr(>F) |  |
| SiteGrassblok 2 651.1 325.6 1.673 0.221 |  |
| Residuals 15 2918.8 94.6 |  |
|  |  |
| BLOCK FF - ALL ANCIENT SHRUBS | NS |
| Df Sum Sq Mean Sq F value Pr(>F) |  |
| SiteShrublok 2 127.0 63.49 1.114 0.354 |  |
| Residuals 15 854.5 56.97 |  |
|  |  |
| BLOCK FF - ALL MODERN SHRUBS | NS |
| Df Sum Sq Mean Sq F value Pr(>F) |  |
| SiteShrublok 2 22.25 11.124 1.726 0.211 |  |
| Residuals 15 96.65 6.443 |  |
|  |  |
| BLOCK FF - ALL SHRUBS | NS |
| Df Sum Sq Mean Sq F value Pr(>F) |  |
| SiteShrublok 2 47.82 23.91 1.451 0.266 |  |
| Residuals 15 247.27 1 6.48 |  |
|  |  |
| BLOCK FF - ALL ANCIENT SMALL TREES | Tukey multiple comparisons of means |
| Df Sum Sq Mean Sq F value Pr(>F) | 95% family-wise confidence level |
| SiteSTblok 2 5.796 2.8978 3.558 0.0544 . | $SiteSTblok |
| Residuals 15 12.215 0.8143 | diff lwr upr p adj |
|  | B-A -0.4166667 -1.769956319 0.936623 0.7088621 |
| Signif. codes: 0 ‘***’ 0.001 ‘**’ 0.01 ‘*’ 0.05 ‘.’ 0.1 ‘ ’ 1 | C-A 0.9400000 -0.413289652 2.293290 0.2018370 |
|  | C-B 1.3566667 0.003377015 2.709956 0.0493911 |
|  |  |
| BLOCK FF - ALL MODERN SMALL TREES | NS |
| Df Sum Sq Mean Sq F value Pr(>F) |  |
| SiteSTlok 2 0.463 0.2313 0.709 0.508 |  |
| Residuals 15 4.894 0.3263 |  |
|  |  |
| BLOCK FF ALL SMALL TREES | Tukey multiple comparisons of means |
| Df Sum Sq Mean Sq F value Pr(>F) | 95% family-wise confidence level |
| SiteSTblok 2 1.914 0.9572 3.942 0.0421 * | $SiteSTblok |
| Residuals 15 3.642 0.2428 | diff lwr upr p adj |
|  | B-A -0.06416667 -0.80314961 0.6748163 0.9723984 |
| Signif. codes: 0 ‘***’ 0.001 ‘**’ 0.01 ‘*’ 0.05 ‘.’ 0.1 ‘ ’ 1 | C-A 0.65750000 -0.08148294 1.3964829 0.0849576 |
|  | C-B 0.72166667 -0.01731628 1.4606496 0.0560689 |
| BLOCK FF TALL TREES ANCIENT | NS |
| Df Sum Sq Mean Sq F value Pr(>F) |  |
| TTSiteblok 2 0.184 0.09201 0.425 0.662 |  |
| Residuals 15 3.249 0.21660 |  |
|  |  |
| BLOCK FF TALL TREES MODERN | NS |
| Df Sum Sq Mean Sq F value Pr(>F) |  |
| TTSiteblok 2 0.01701 0.008506 1.386 0.28 |  |
| Residuals 15 0.09203 0.006136 |  |
|  |  |
| BLOCK FF TALL TREES ALL | NS |
| Df Sum Sq Mean Sq F value Pr(>F) |  |
| TTSiteblok 2 0.0734 0.03670 0.564 0.58 |  |
| Residuals 15 0.9759 0.06506 |  |
| NON-VEGETATION |  |
| BLOCK FF BAREGROUND | NS |
| Df Sum Sq Mean Sq F value Pr(>F) |  |
| BGsite 2 79.1 39.53 0.459 0.641 |  |
| Residuals 15 1292.0 86.13 |  |
|  |  |
| BLOCK FF LEAF LITTER | NS |
| Df Sum Sq Mean Sq F value Pr(>F) |  |
| LSsite 2 1638 818.9 2.58 0.109 |  |
| Residuals 15 4762 317.4 |  |
|  |  |
| BLOCK FF SHADE | NS |
| Df Sum Sq Mean Sq F value Pr(>F) |  |
| LSsite 2 1.109 0.5543 1.393 0.279 |  |
| Residuals 15 5.970 0.3980 |  |

TABLE S3d: Grass coverage, treatment, origin and life strategy
ANOVA: grass coverage ~ treatment, x origin (Sunda-Sahul), x life strategy (annual v perennial).

| Effect | SS | df | MS | F | p |
| --- | --- | --- | --- | --- | --- |
| Treatment | 1756.80 | 5 | 351.36 | 1.25 | 0.301 |
| Origin | 2150.86 | 1 | 2150.86 | 7.65 | 0.008 |
| Lifestrategy | 9160.75 | 1 | 9160.75 | 32.59 | <0.001 |
| Treatment × Origin | 5073.50 | 5 | 1014.70 | 3.61 | 0.007 |
| Treatment × Lifestrategy | 238.10 | 5 | 47.62 | 0.17 | 0.973 |
| Origin × Lifestrategy | 0.55 | 1 | 0.55 | 0.002 | 0.965 |
| Treatment × Origin × Lifestrategy | 1469.37 | 5 | 293.87 | 1.05 | 0.402 |
| Residual | 13493.79 | 48 | 281.12 | — | — |

TABLE S3e: Block effect on grass coverage.

ANOVA: Modern annual grasses (% coverage) ~ treatment
Subset: Biogeographic group **= modern, Life strategy = annual.**)

| Effect | df | F | p |
| --- | --- | --- | --- |
| Treatment | 5 | 0.77 | **0.591** |
| Residuals | 12 |  |  |

### ANOVA: Modern annual grasses % coverage ~ block Subset: Biogeographic group **= modern, Lifestrategy = annual.**

| Effect | df | F | p |
| --- | --- | --- | --- |
| **Block** | 2 | **5.06** | **0.021** |
| Residual | 15 |  |  |

### Tukey post-hoc comparisons

| Comparison | Difference | p |
| --- | --- | --- |
| **A vs B** | 25.14 | 0.048 |
| **A vs C** | 27.53 | 0.030 |
| B vs C | 2.39 | 0.966 |

### Mean response by block (% grass coverage)

| A | 32.64 |
| --- | --- |
| B | 7.50 |
| C | 5.11 |

TABLE S3f: Biogeographical group–life strategy-treatment

Tukey HSD pairwise comparisons: treatments, with biogeographical group / life strategy combinations.
Life strategy: A=Annual, P= Perennial

| Biogeograpical group | Life strategy | Comparison | Mean difference | p-adj |
| --- | --- | --- | --- | --- |
| Ancient | A | E1 – E3 | 6.08 | 0.704 |
| Ancient | A | E3 – E5 | −4.49 | 0.886 |
| Ancient | P | E1 – E5 | 22.05 | 0.486 |
| Ancient | P | E5 – L2 | −15.62 | 0.783 |
| Modern | A | E1 – UB | −22.81 | 0.762 |
| Modern | A | L2 – UB | −25.40 | 0.678 |
| Modern | P | **E1 – E5** | **−52.50** | **0.082** |
| Modern | P | E1 – UB | −46.41 | 0.145 |

**Notes**

- Values are **Tukey-adjusted p-values**.
- No pairwise treatment comparisons were statistically significant at α = 0.05.
- The largest difference occurred between **E1 and E5 for Modern perennials**.

## Appendix S4 Sprouts

TABLE S4a: Sprouts—Mean abundance for each species, in each treatment.

Includes only species which had a mean of at least 1 individual per 100 m^2^, according to fire frequency.
 * denotes fully deciduous species

|  | Ancient | | | Modern | |
| --- | --- | --- | --- | --- | --- |
| Treatment | Species | Mean abundance / 100 m^2^ | | Species | Mean abundance / 100 m^2^ |
|  | *Acacia spp* | 3.8 | | *Buchanania** | 1.7 |
| UB | *Persoonia* | 3.0 | |  |  |
|  | *Syzygium* | 1.7 | |  |  |
|  | *Eucalyptus* | 1.6 | |  |  |
|  | *Calytrix spp* | 1.1 | |  |  |
| E5 | *Calytrix spp* | 3.4 | | *Buchanania** | 2.4 |
|  | *Persoonia* | 2.6 | |  |  |
|  | *Eucalyptus* | 2.3 | |  |  |
|  | *Syzygium spp* | 1.8 | |  |  |
|  | *Acacia spp* | 1.6 | |  |  |
|  | *Petalostigma spp* | 1.0 | |  |  |
| E3 | *Persoonia* | 3.7 | | *Buchanania** | 7.1 |
|  | *Eucalyptus* | 3.1 | | *Coelospermum* | 4.5 |
|  | *Calytrix spp* | 2.5 | | *Gardenia spp* | 2.3 |
|  | *Acacia spp* | 2.1 | | *Erythrophleum* | 1.4 |
|  | *[Syzygium spp* | 0.9] | | *Terminalia** | 1.1 |
|  |  |  | | *Pachynema (Hibbertia)* | 1.0 |
|  |  |  | | *Planchonia** | 0.9] |
| E2 | *Syzygium spp* | 8.7 | | *Buchanania** | 6.5 |
|  | *Eucalyptus spp* | 4.4 | | *Coelospermum* | 5.9 |
|  | *Persoonia* | 4.1 | | *Gardenia spp* | 1.4 |
|  | *Grevillea* | 4.1 | | *Pachynema* | 1.0 |
| L2 | *Persoonia* | 2.8 | | *Buchanania** | 5.4 |
|  | *Syzygium spp* | 1.5 | | *Coelospermum* | 1.3 |
|  | *Eucalyptus spp* | 1.5 | | *Terminalia** | 1.0 |
| E1 | *Eucalyptus spp* | 4.8 | | *Coelospermum* | 5.3 |
|  | *Acacia spp* | 3.7 | | *Buchanania** | 2.2 |
|  | *Persoonia* | 1.1 | | *Pachynema* | 1.2 |
|  |  |  | | *Planchonia* | 1.2 |
|  |  | |  | *Erythrophleum* | 1.0 |

FIGURE S4b: Shift in mean sprout abundance (per 100 m^2^) according to fire frequency.

Pale colour = % change from Low to Mid FF (E2-E3).
Dark colour = % change from Low to High FF (E1-L2).
Grey = species present as sprouts only in Mid & High FF sites, % change from Mid to HIgh FF.

* denotes fully deciduous species. ** note: the extremely high number of sprouts in Mid FF sites was due almost entirely due to mass sprouting of *Grevillea pteridifolia* on one E2 site (B5).

This chart compares mean abundance change from base point (0%) in Low FF (UB-E5) sites. Note that the scale is set in 25% increments from 0%–800+%.

For example, mean sprout abundance in *Melaleuca*: in Mid FF sites (pale block), sprouts were 25% more abundant than in the Low FF sites; in High FF sites (dark block), sprouts were 50% more abundant than in Low FF sites.

## Appendix S5 Shrubs

FIGURE S5a: Top 5 most abundant shrubs in each origin type.
Note that 4 of the 5 modern species are fully dry-season deciduous*.


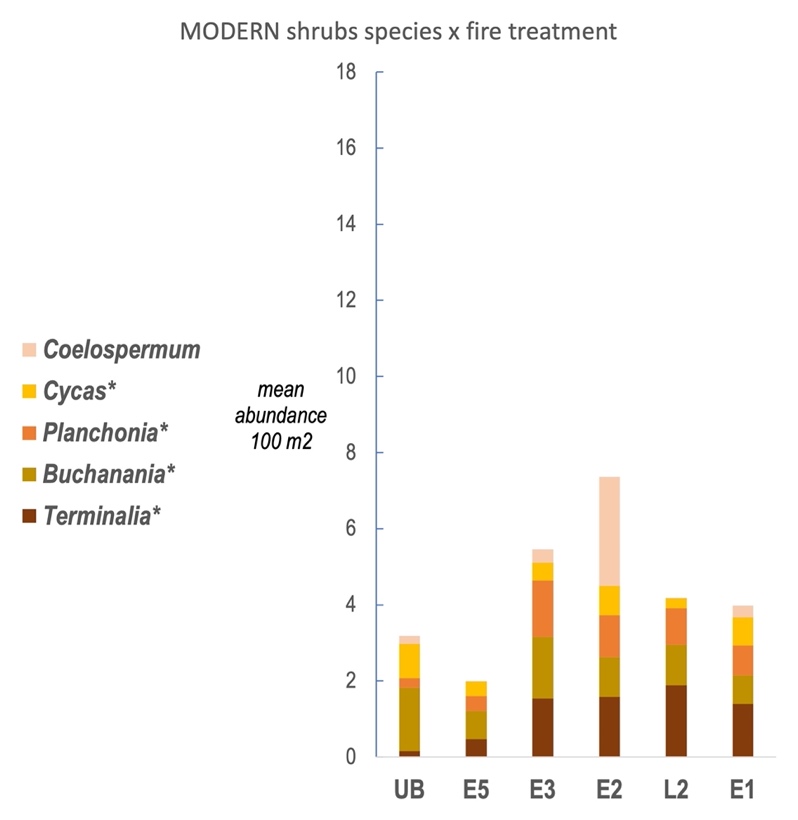

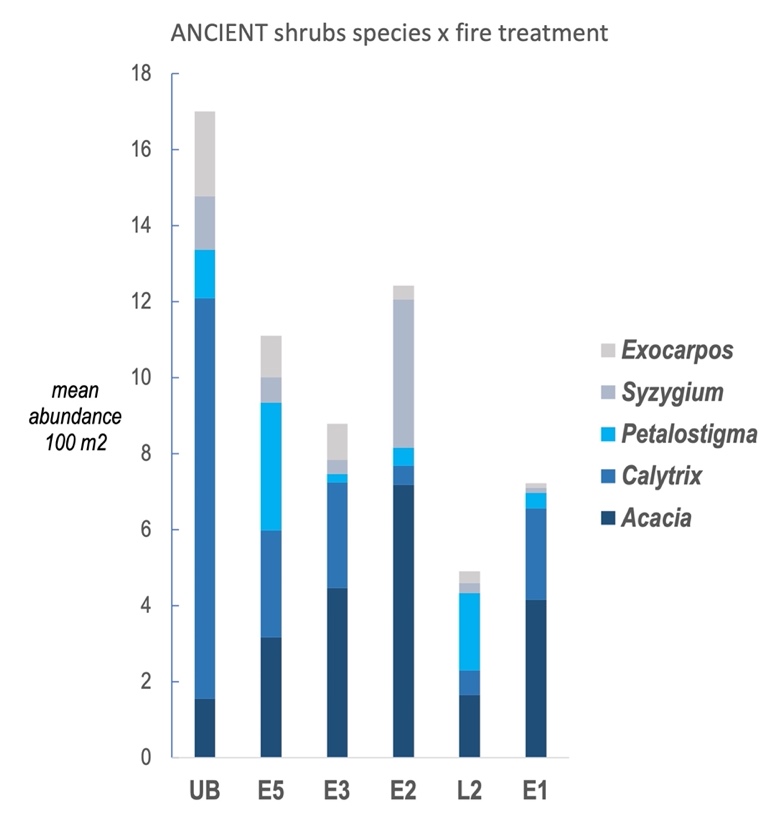


FIGURE SI-5b: Abundance of deciduous trees and shrubs in relation to fire frequency.
Note that all of these are Sundanian genera: *Terminalia, Planchonia, Buchanania, Erythrina and Cycas*.


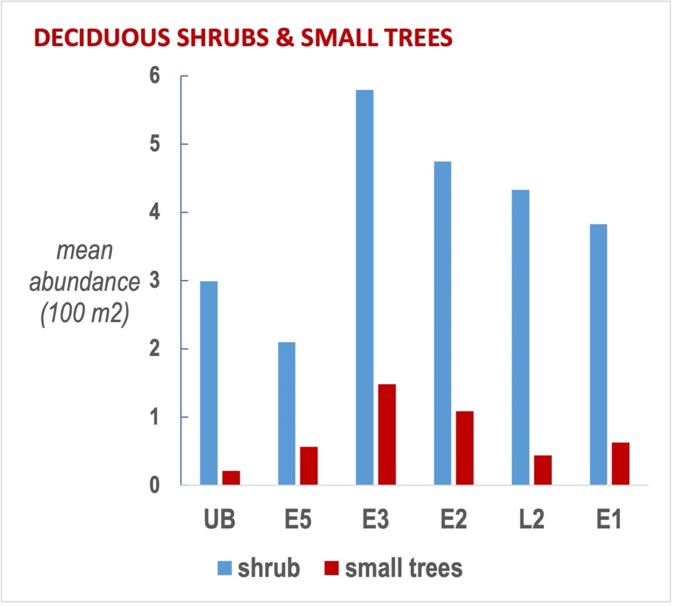


## Appendix S6 Small trees

FIGURE S6a: Species richness in small trees by origin throughout fire-frequency treatments.
Orange = presence. * = fully deciduous

| **SAHUL** | *species* | | **UB** | | **E5** | | **E3** | | **E2** | | **L2** | | **E1** | |
| --- | --- | --- | --- | --- | --- | --- | --- | --- | --- | --- | --- | --- | --- | --- |
| *Acacia* | *aurelius* | | x | | x | | 0 | | x | | 0 | | 0 | |
| *Acacia* | *holosericea* | | 0 | | x | | x | | 0 | | 0 | | 0 | |
| *Acacia* | *lamprocarpa* | | x | | x | | x | | x | | x | | x | |
| *Acacia* | *mimulus* | | x | | x | | x | | 0 | | 0 | | 0 | |
| *Acacia* | *platycarpa* | | 0 | | x | | x | | x | | 0 | | x | |
| *Acacia* | *latescens* | | 0 | | 0 | | 0 | | x | | 0 | | x | |
| *Alphitonia* | *excelsa* | | x | | x | | x | | x | | x | | x | |
| *Banksia* | *dentata* | | 0 | | 0 | | 0 | | x | | 0 | | 0 | |
| *Calytrix* | *brownii, exstipulata* | | 0 | | x | | x | | 0 | | 0 | | 0 | |
| *Denhamia* | *obscura* | | x | | 0 | | 0 | | 0 | | 0 | | 0 | |
| *Corymbia* | *bleeseri* | | x | | x | | x | | x | | x | | x | |
| *Eucalyptus* | *miniata* | | x | | x | | x | | x | | x | | x | |
| *Eucalyptus* | *tetradonta* | | x | | x | | x | | x | | x | | x | |
| *Exocarpos* | *latifolius* | | x | | 0 | | 0 | | 0 | | 0 | | 0 | |
| *Grevillea* | *goodii, pteridifolia* | | x | | x | | x | | x | | 0 | | x | |
| *Melaleuca* | *viridiflora* | | x | | x | | 0 | | 0 | | 0 | | x | |
| *Persoonia* | *falcata* | | x | | x | | x | | x | | x | | x | |
| *Petalostigma* | *pubescens, quadricloculare* | | x | | x | | x | | x | | x | | x | |
| *Stenocarpus* | *acacioides* | | x | | 0 | | 0 | | 0 | | 0 | | 0 | |
| *Syzygium* | *suborbiculare* | | x | | x | | x | | x | | 0 | | 0 | |
| *Syzygium* | *eucalyptoides* | | x | | x | | x | | x | | 0 | | 0 | |
| *Xanthostemon* | *parodoxus* | | x | | x | | x | | x | | x | | x | |
| **species richness** | | **17** | | **17** | | **15** | | **15** | | **8** | | **12** | |  |

| **SUNDA** | *species* | | **UB** | **E5** | **E3** | **E2** | **L2** | **E1** |
| --- | --- | --- | --- | --- | --- | --- | --- | --- |
| *Livistona* | *humilis* | | 0 | x | x | 0 | 0 | xx |
| *Alstonia* | *actinophylla* | | 0 | x | 0 | 0 | 0 | 0 |
| *Buchanania ** | *obovata* | | x | x | x | x | 0 | xx |
| *Canarium* | *australianum* | | x | x | 0 | x | 0 | 0 |
| *Carallia* | *brachiata* | | x | 0 | 0 | 0 | 0 | 0 |
| *Cycas ** | *armstrongii* | | xx | x | x | x | 0 | xx |
| *Erythrina ** | *variegata v.orientalis* | | 0 | 0 | x | x | 0 | 0 |
| *Erythrophleum* | *chlorostachys* | | x | 0 | x | x | x | xx |
| *Gardenia* | *schwarzii, megasperma* | | x | x | x | x | x | xx |
| *Pandanus* | *spiralis* | | x | x | 0 | x | 0 | 0 |
| *Planchonia* | *careya* | | x | x | x | x | x | xx |
| *Terminalia ** | *ferdinandiana* | | 0 | x | x | x | x | xx |
| *Vitex* | *glabrata* | | 0 | 0 | 0 | 0 | 0 | xx |
| *Ficus* | *aculeata* | | 0 | 0 | 0 | 0 | 0 | xx |
| **species richness** | |  | **8** | **9** | **8** | **9** | **4** | **9** |

FIGURE S6b: Small tree abundance, species composition and distribution of main genera in relation to fire frequency and species origin.

| **SAHULIAN** |  |
| --- | --- |
| 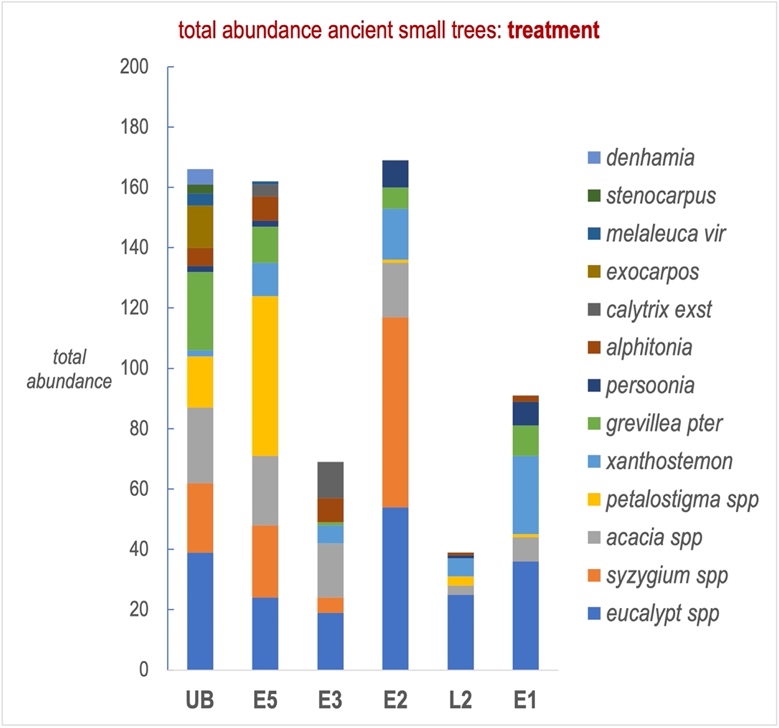 |  |
| **SUNDANIAN** * = fully deciduous |  |
| 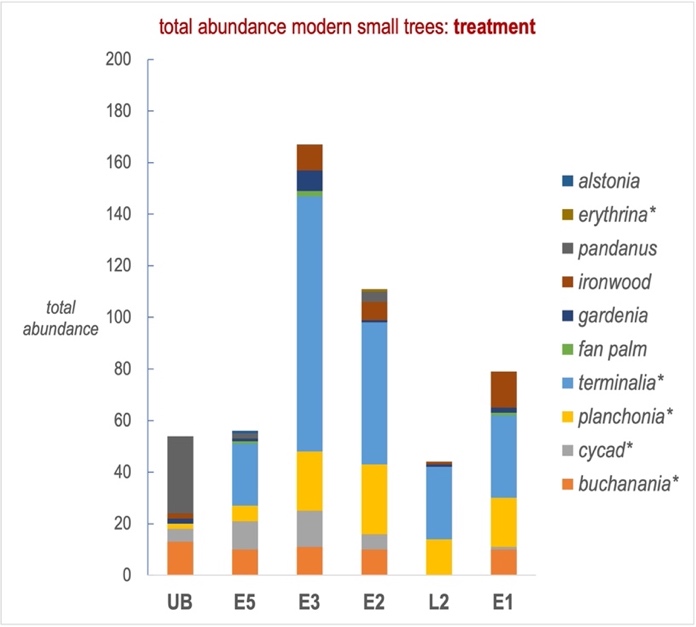 |  |
|  |  |
|  |  |

## Appendix S7 Summary of mean abundance across the whole site

TABLE S7: Abundance and proportion of stems in each non-grass stratum.
Mean abundance of stems per 100 m^2^ (± 1 SE).

| Treatment | Grasses | Sprouts | % | Shrubs | % | Small  trees | % | Tall  trees | % | Total % |
| --- | --- | --- | --- | --- | --- | --- | --- | --- | --- | --- |
| UB | 54.43 (6.4) | 15.04 (1.9) | 33.75 | 26.54 (4.6) | 59.57 | 2.33 (0.3) | 5.24 | 0.65 (0.2) | 1.45 | 100.00 |
| E5 | 61.78 (8.2) | 19.06 (2.6) | 49.93 | 15.90 (2.7) | 41.66 | 2.43 (0.5) | 6.38 | 0.78 (0.2) | 2.04 | 100.00 |
| E3 | 85.92 (9.8) | 33.46 (3.2) | 60.09 | 18.87 (1.0) | 33.89 | 2.37 (0.3) | 4.26 | 0.98 (0.2) | 1.76 | 100.00 |
| E2 | 85.97 (8.9) | 45.90 (4.5) | 62.36 | 22.95 (3.0) | 31.17 | 3.05 (0.4) | 4.14 | 1.71 (0.4) | 2.33 | 100.00 |
| L2 | 107.02 (13.69) | 20.37 (1.5) | 61.23 | 11.60 (1.5) | 34.86 | 0.87 (0.2) | 2.61 | 0.43 (0.1) | 1.30 | 100.00 |
| E1 | 107.39 (17.22) | 23.34 (4.1) | 55.81 | 15.79 (2.4) | 37.77 | 1.76 (0.2) | 4.22 | 0.92 (0.2) | 2.20 | 100.00 |

## Appendix S8 Bare ground, leaf litter and shade

FIGURE S8. Effect of fire frequency on (A) bare ground and leaf litter, and (B) shade,
showing mean (± 1SE) proportion of plots occupied. Shade scores: 5 = 100% shade, 1= full sun.


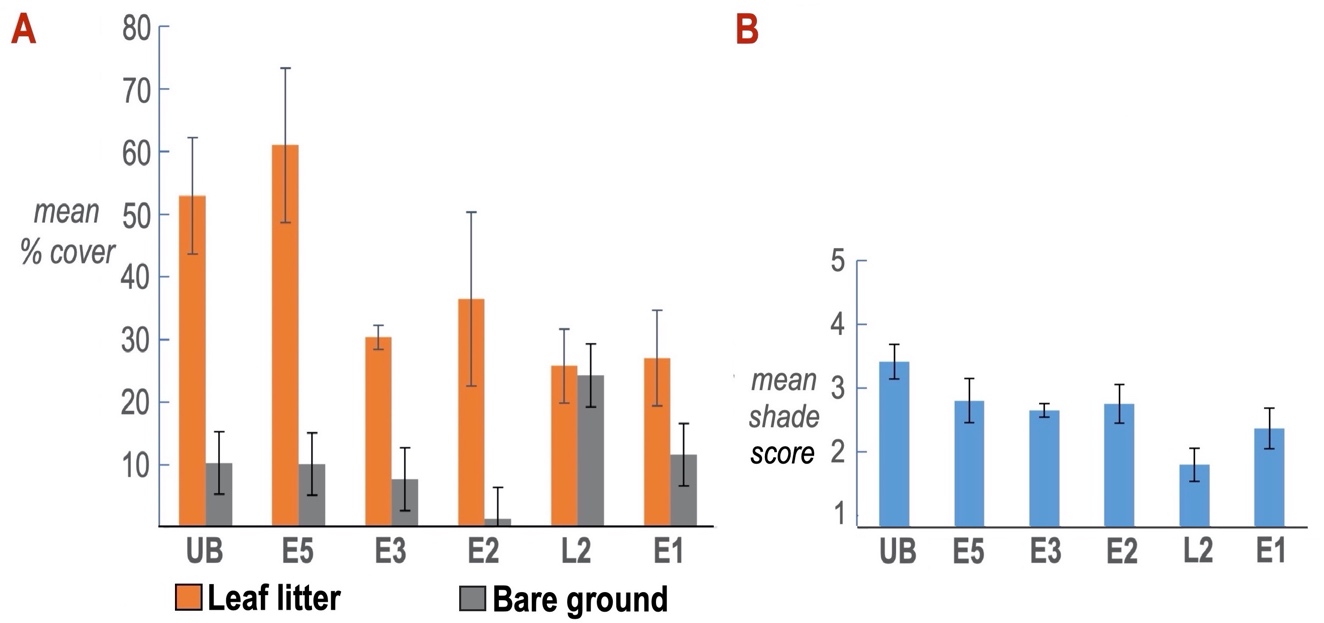


## Appendix S9: Relationship between lightning and fire in northern Australia

Figure S9: Comparison of mean monthly distribution of lightning incidence and fire-affected areas across northern Australian regions.

The mismatch of lighting-intensive months and fire-intensive months shows that lightning is not the cause of most fires. Adapted from Russell-Smith et al (2007). Lightning incidence = red bars. Fire-affected area = solid black line. % = % of fire-affected area, and % of lightning.


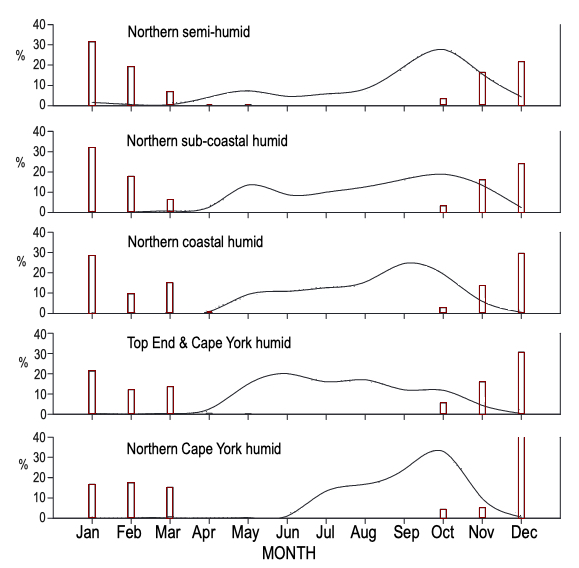

Supplement: Supplementary file 1 — Appendix S1: Species list. Appendix S2: Quadrats surveyed. Appendix S3: ANOVA summaries & detail. Appendix S4: Sprouts. Appendix S5: Shrubs. Appendix S6: Small trees. Appendix S7: Summary of mean abundance across the whole site. Appendix S8: Bare ground, leaf litter and shade. Appendix S9: Relationship between lightning and fire in northern Australia. [file ECE3-16-e73837-s001.docx]
